# Evaluating surgical strategies for pediatric congenital choledochal cysts: a multicenter retrospective study and network meta-analysis

**DOI:** 10.3389/fped.2025.1678421

**Published:** 2025-09-26

**Authors:** Chuang Cao, Zhibin Xu, Long Cen, Tianfu Mai, Jihuang Huang, Chuan Tian

**Affiliations:** ^1^ Department of Pediatric Surgery, Affiliated Hospital of Guangdong Medical University, Zhanjiang, Guangdong, China; ^2^ Departments of Organ Transplantation, First Affiliated Hospital of Guangzhou Medical University, Guangzhou, Guangdong, China; ^3^ Department of Pediatric Surgery, Guangdong Maternal and Child Health Hospital, Guangzhou, Guangdong, China; ^4^ Pediatric Medicine Center, Affiliated Hospital of Guangdong Medical University, Zhanjiang, Guangdong, China

**Keywords:** congenital choledochal cyst, network meta-analysis, robotic-assisted surgery, laparoscopic surgery, pediatric surgical outcomes, Bayesian model

## Abstract

**Objective:**

This study compared the efficacy and safety of open, laparoscopic, and robotic-assisted surgeries for pediatric congenital choledochal cysts (CCC) using network meta-analysis, with retrospective cohort data to validate findings.

**Methods:**

Following the PRISMA guidelines, 28 cohort studies involving a total of 3,672 patients were included. Key outcomes assessed included operative time, hospital stay, intraoperative blood loss, postoperative bile leakage rate, and postoperative bowel obstruction rate. A Bayesian model was employed for the network meta-analysis, with heterogeneity and consistency checks as well as publication bias assessments. Furthermore, a retrospective cohort study was conducted on 72 CCC patients who underwent surgery between January 2010 and January 2025 at two medical centers [60 cases in the open surgery group [OSG] and 12 cases in the laparoscopic surgery group [LSG]]. These data were incorporated into the meta-analysis to evaluate consistency with prior findings.

**Results:**

The 28 studies (2007–2025) included two three-arm and 26 two-arm studies. Newcastle-Ottawa Scale assessment identified biases in selection and follow-up in some studies. Open surgery had the shortest operative time (MD = −1.101 vs. laparoscopic, 95% CI: −1.368 to −0.834; MD = −1.39 vs. robotic, 95% CI: −1.69 to −1.09), followed by robotic-assisted, then laparoscopic surgery. Robotic-assisted surgery had the shortest hospital stay (MD = −1.98 vs. open, 95% CI: −2.72 to −1.19), followed by laparoscopic. Laparoscopic surgery had the least blood loss (MD = 46.76 vs. open, 95% CI: 10.36–83.64), followed by robotic-assisted. Robotic-assisted surgery had the lowest bile leakage rate; laparoscopic had the lowest bowel obstruction rate (OR = 0.11 vs. open, 95% CI: 0.01–0.6). Retrospective data showed OSG had shorter operative time (3.52 ± 0.82 vs. 5.61 ± 1.24 h, *P* < 0.01), longer hospital stays (15.98 ± 4.99 vs. 12.92 ± 2.15 days, *P* < 0.05), and greater blood loss (90.45 ± 62.29 vs. 46.00 ± 26.52 ml, *P* < 0.05) than LSG, with no significant difference in complications. Updated meta-analysis confirmed consistent rankings.

**Conclusions:**

Robotic-assisted surgery excels in reducing hospital stay and bile leakage, laparoscopic surgery minimizes blood loss and bowel obstruction, while open surgery is fastest but inferior in other outcomes. These findings guide CCC surgical decisions, though randomized trials are needed.

## Introduction

Congenital choledochal cyst (CCC) is a rare but significant biliary malformation in children, characterized by cystic or fusiform dilatation of the common bile duct, often accompanied by intrahepatic bile duct dilatation (1). The underlying pathogenesis of CCC remains unclear. Clinical manifestations of CCC are diverse, including jaundice, abdominal pain, an upper abdominal mass, and fever, all of which significantly impair the quality of life and growth of affected children. Studies report an incidence of approximately 1 in 150,000 live births in the United States, whereas in East Asian countries, particularly China and Japan, the incidence is markedly higher, at 1 in 13,000 (2). Without timely intervention, CCC can lead to recurrent biliary tract infections, pancreatitis, bile leakage, and, in severe cases, bile duct malignancies or biliary cirrhosis, posing a substantial threat to the patient's life. Therefore, early diagnosis and effective treatment are critical for improving outcomes.

Surgical resection remains the gold standard for CCC treatment. The preferred approach involves cyst excision and Roux-en-Y hepaticojejunostomy, aiming to eliminate the lesion and reconstruct bile flow. With advances in surgical techniques, treatment options have evolved from traditional open surgery to minimally invasive approaches, including laparoscopic and robot-assisted procedures. Open surgery, being the most established technique, is widely performed due to its simplicity and effectiveness. However, driven by the need for reduced postoperative scarring and faster recovery, laparoscopic surgery has gained traction since its first application to CCC in 1995 (3). In 2006, Woo et al. (4) successfully utilized the da Vinci robotic surgical system for CCC management, demonstrating its safety and feasibility. Compared to laparoscopic surgery, the da Vinci system offers enhanced precision and dexterity, showing promise as a potential alternative. To account for variations in robotic platforms (e.g., da Vinci models S/Si/Xi/SP), we extracted and analyzed available manufacturer and model information in subgroup analyses.

The hallmark of CCC is cystic dilatation of intrahepatic or extrahepatic bile ducts. Approximately 80% of cases are diagnosed prenatally or during infancy. Complete cyst excision and biliary reconstruction are typically required for treatment, with Roux-en-Y hepaticojejunostomy and hepaticoduodenostomy being the main reconstructive options. To address potential influences of reconstruction type on outcomes, we performed subgroup analyses by hepaticojejunostomy vs. hepaticoduodenostomy where data allowed.

Despite being the first-line treatment for CCC, the relative efficacy and safety of different surgical approaches remain contentious. For instance, a retrospective cohort study by Xie et al. (5) showed that laparoscopic surgery required significantly longer operative times than both open and robot-assisted surgeries but resulted in shorter hospital stays and faster recovery. Similarly, Kim et al. (6) reported that although the intraoperative blood loss in robot-assisted surgery was higher than in open surgery, the hospitalization duration was comparable. In contrast, Lee et al. (7) observed similar hospital stays for open and laparoscopic surgeries but noted significant differences in postoperative complication rates. Domestic studies have also contributed to this field: Xie Xiaolong et al. (8) found that both laparoscopic and robot-assisted procedures resulted in shorter hospital stays and faster recovery than open surgery. Additionally, Chi et al. (9) demonstrated that robot-assisted surgery was associated with significantly lower blood loss and complication rates compared to laparoscopic surgery. Furthermore, a systematic review by Sun et al. (10) highlighted the advantages of laparoscopic surgery in reducing the incidence of long-term postoperative complications. However, discrepancies persist regarding outcomes such as bile leakage and intestinal obstruction, underscoring the need for more comprehensive analyses.

To address these issues, this study employs evidence-based medicine methods to systematically evaluate the efficacy and safety of open, laparoscopic, and robot-assisted surgeries for CCC through a Bayesian network meta-analysis. The primary outcomes include operative time, hospital stay, and intraoperative blood loss, while secondary outcomes cover postoperative bile leakage and intestinal obstruction rates. Additionally, we collected multicenter clinical data on CCC patients undergoing open (open cyst excision and Roux-en-Y hepaticojejunostomy) or laparoscopic surgery (laparoscopic cyst excision and Roux-en-Y hepaticojejunostomy) between January 1, 2010, and January 1, 2025, at Guangdong Medical University Affiliated Hospital and Guangzhou Women and Children's Medical Center. These clinical data were incorporated into the meta-analysis to assess whether the results remained consistent before and after their inclusion.

This study aims to provide high-quality evidence to inform the optimal surgical management of CCC, while addressing the clinical diversity and complexity of this condition. The findings are expected to contribute significantly to the refinement of CCC treatment strategies and guidelines.

## Materials and methods

The network meta-analysis component of this study was conducted in accordance with the Preferred Reporting Items for Systematic Reviews and Meta-Analyses (PRISMA) guidelines (11), ensuring a systematic and transparent approach to data synthesis and reporting. The study protocol was registered with PROSPERO, an international register of systematic reviews, under registration ID CRD42019137474.

For the retrospective cohort analysis, clinical data were collected from children diagnosed with congenital choledochal cyst (CCC) who underwent surgical treatment at Guangdong Medical University Affiliated Hospital and Guangzhou Women and Children's Medical Center between January 1, 2010, and January 1, 2025. A total of 72 patients met the inclusion criteria, with 60 children who underwent open surgery assigned to the open surgery group (OSG) and 12 children who underwent laparoscopic surgery assigned to the laparoscopic surgery group (LSG). The baseline characteristics of the two groups, including age, gender, and weight, were well-balanced, with no statistically significant differences observed.

This study received ethical approval from the Ethics Committee of Guangdong Medical University Affiliated Hospital (Approval No. KY20241121) and was registered on ClinicalTrials.gov (Registration No. NCT2034334) (Supplementary Figure S1).

### Search strategy and data sources

We conducted a comprehensive search of both Chinese and international databases. The Chinese databases included China National Knowledge Infrastructure (CNKI), Wanfang Data, and the Chinese Biomedical Literature Database (CBM). The international databases searched were PubMed, EMBASE, Web of Science, and the Cochrane Library. Search terms included “choledochal cyst” “open” “laparoscopy” “robotic surgical procedures” “operative surgical procedures,” and related keywords. These terms were combined using Boolean operators (AND, OR, NOT) to refine the search.

For CBM, PubMed, EMBASE, Web of Science, and the Cochrane Library, we employed a combination of free-text and MeSH/subject terms. For CNKI, Wanfang Data, and Web of Science, a professional search strategy tailored to these platforms was utilized. The detailed search strategy is available on Zenodo: Cao, C., & Huang, J. (2024). *Comparative Analysis of Surgical Techniques for Pediatric Congenital Choledochal Cysts: A Network Meta-analysis*. Zenodo. https://doi.org/10.5281/zenodo.14194806.

### Eligibility criteria

#### Inclusion criteria for the network meta-analysis

a. Study Population: Pediatric patients.
b. Treatment Methods: The study must include at least two of the following three treatment methods:
c. Open surgery (open cyst excision and Roux-en-Y hepaticojejunostomy),
d. Laparoscopic surgery (laparoscopic cyst excision and Roux-en-Y hepaticojejunostomy),
e. Da Vinci robotic-assisted surgery (robotic cyst excision and Roux-en-Y hepaticojejunostomy).
f. Outcome Measures:
  1. **Primary Outcomes**: Surgery time, hospital stay, and intraoperative blood loss.
  2. **Secondary Outcomes**: Incidence of postoperative bile leakage and intestinal obstruction.

#### Exclusion criteria for the network meta-analysis

a. Studies with no measurable outcomes or outcomes that cannot be calculated.
b. Editorials or commentaries.
c. Duplicate publications.

#### Inclusion criteria for the retrospective study

a. Pediatric patients diagnosed with congenital choledochal cyst based on preoperative imaging.
b. Treatment methods included either:

 1. Open surgery (open cyst excision and roux-en-Y hepaticojejunostomy), or
 2. Laparoscopic surgery (laparoscopic cyst excision and Roux-en-Y hepaticojejunostomy).
  c. Informed consent for follow-up was obtained from the patient's family.

#### Exclusion criteria for the retrospective study

a. Patients lacking one or more key outcome measures, including surgery time, hospital stay, intraoperative blood loss, incidence of postoperative bile leakage, or incidence of postoperative intestinal obstruction.
b. Patients with incomplete clinical data, such as missing sex or age information.
c. Patients with coagulation disorders.
d. Patients with severe comorbidities or immune system disorders.

### Data extraction and risk of bias assessment

#### Literature screening and risk of bias assessment

The search results from all databases were imported into EndNote X9 (Clarivate Analytics, Philadelphia, PA, USA) to remove duplicate records. Two researchers independently reviewed the included studies, designed a data extraction form, and extracted relevant information based on the form. Extracted data included the author, publication year, treatment methods, gender, age, weight, study type, and outcome measures (surgery time, hospital stay, intraoperative blood loss, postoperative bile leakage incidence, and postoperative intestinal obstruction). Any disagreements were resolved through discussion or consultation with a third researcher.

The risk of bias was assessed using the Newcastle-Ottawa Scale (NOS) (12), which evaluates the quality of cohort studies across three domains: selection of study populations, comparability of groups, and assessment of exposure or outcome. The scale consists of the following eight items:

a. Representativeness of the exposed group;
b. Selection of the non-exposed group;
c. Ascertainment of exposure;
d. Demonstration that the outcome of interest was not present at the start of the study;
e. Comparability of cohorts based on the design or analysis (this item has a maximum score of 2, while others have a maximum score of 1);
f. Adequacy of outcome assessment;
g. Adequacy of follow-up duration;
h. Completeness of follow-up for both exposed and non-exposed groups.

The NOS uses a star-based semi-quantitative system to assess the quality of studies, with a maximum score of 9.

### Surgical procedures and evaluation metrics for the retrospective study

Detailed surgical protocols for open and laparoscopic procedures, including preoperative preparation, intraoperative steps, and postoperative care, are provided in the Supplementary Material. The collected metrics included gender, age, weight, admission temperature, surgical method, surgery duration, intraoperative blood loss, conversion-to-open-surgery rate, hospital stay, incision infection rate, postoperative bile leakage rate, and postoperative intestinal obstruction rate (Supplementary Figure S1).

### Statistical analysis

The network meta-analysis in this study was performed using Stata 14.0 (StataCorp LLC, College Station, TX, USA). If closed loops were present, mixed treatment effect analysis was applied; otherwise, adjusted indirect comparison analysis was used. The “networkplot” command in Stata was used to generate network diagrams, where nodes represent different treatment methods, lines indicate direct comparisons, and the size of nodes and the thickness of lines reflect the sample size and the number of studies. Based on various outcome measures, surface under the cumulative ranking (SUCRA) values were calculated for different surgical methods to determine their rankings.

Heterogeneity among studies was assessed using the *I*^2^ statistic. When *I*^2^ >50%, a random-effects model was applied. For continuous variables such as surgery time, hospital stay, and intraoperative blood loss, weighted mean differences (MD) with 95% confidence intervals (CI) were calculated. For categorical variables such as postoperative bile leakage rate and postoperative intestinal obstruction rate, odds ratios (OR) with 95% CI were calculated. Consistency testing was performed using node analysis to evaluate inconsistencies between direct and indirect comparison results. If no inconsistency was detected, the network meta-analysis results were used; otherwise, direct comparison results were adopted. Additionally, publication bias was assessed by generating funnel plots using the “netfunnel” command in Stata.

Subgroup analyses were performed by biliary reconstruction type (Roux-en-Y hepaticojejunostomy vs. hepaticoduodenostomy) for primary and secondary outcomes where at least 3 studies per subgroup were available. Meta-regression was used to assess the impact of reconstruction type on effect estimates (e.g., operative time, blood loss), adjusting for study year and sample size. Heterogeneity within subgroups was assessed using I² statistic. If data were insufficient for quantitative synthesis, a descriptive stratification was provided. Sensitivity analyses restricted to studies using Roux-en-Y hepaticojejunostomy were conducted to test robustness. Robotic platform information (manufacturer, model, generation, e.g., da Vinci S/Si/Xi/SP) was extracted from included studies where available. Subgroup analyses were performed by robotic platform type for primary and secondary outcomes when at least 3 studies per subgroup reported details. Meta-regression included platform generation as a covariate, adjusting for study year and sample size, to assess impact on outcomes (e.g., operative time, blood loss). Heterogeneity within subgroups was assessed using *I*^2^. Due to anticipated sparse data, descriptive stratification was provided for platform distribution and qualitative comparison of outcomes. Sensitivity analyses restricted to studies using newer da Vinci models (Xi/SP) were conducted to test robustness. Authors of primary studies were not contacted to clarify platform details due to time constraints. In addition to the meta-analysis, data from the retrospective study were incorporated. Statistical analysis for the retrospective study was conducted using SPSS 22.0 (IBM Corp, Armonk, NY, USA). Continuous variables were presented as mean ± standard deviation (Mean ± SD), and comparisons between groups were performed using independent sample t-tests or corrected t-tests. Categorical variables were expressed as frequencies and percentages, and comparisons were made using continuity-corrected chi-square tests or Fisher's exact tests, with a significance level of *α* = 0.05. After integrating the retrospective study data, the network meta-analysis was re-performed using the abovementioned methods to generate conclusions.

## Result

### Search results and characteristics of included studies

**Figure 1:**
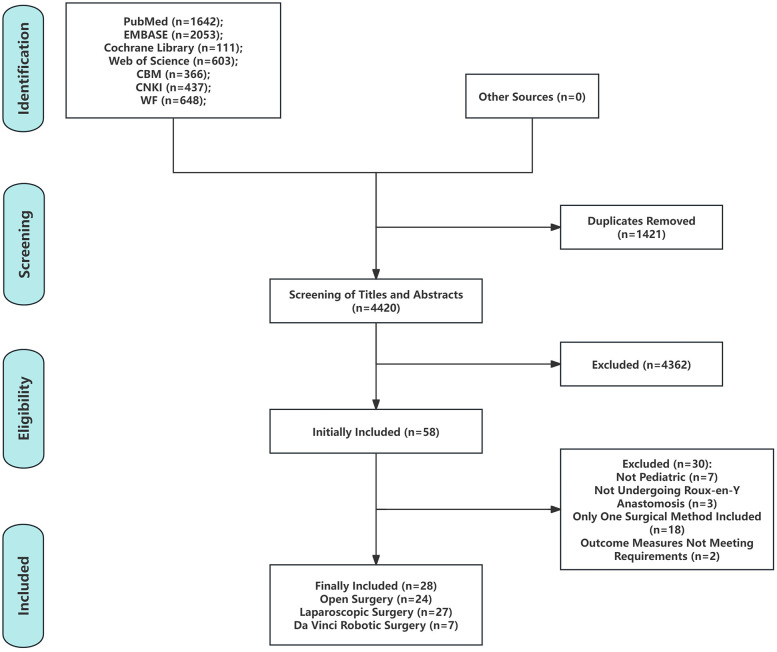
Flow diagram of Literature Screening and Inclusion.

A total of 5,841 articles were identified through a preliminary search of seven Chinese and English databases. After duplicate removal using Endnote X9 (Clarivate Analytics, Philadelphia, PA, USA), 4,420 articles remained. Screening of titles and abstracts yielded 58 articles for full-text review, of which 30 were excluded. Ultimately, 28 studies were included in the analysis (5–9, 13–35) (Figure 1), encompassing 3,672 pediatric patients with publication dates ranging from 2007 to 2025. Of these, 2 studies utilized a three-arm design (5, 8), while the remaining 26 studies were two-arm designs (6, 7, 9, 13–35). Gender distribution was reported in 19 studies (5–9, 14, 16, 17, 19, 20, 22, 24–30, 33), patient age at the time of surgery in 25 studies (5–8, 13–17, 19–35), and patient weight at the time of surgery in 10 studies (5, 7, 20, 21, 23, 25, 26, 28, 30, 31) (Table 1).

**Table 1:** Baseline characteristics of included studies.

Author	Method	*n*	Male	Female	Age (Year, Month, Day)	Weight	Study
Koga et al. (21)	RA	10	NR	NR	5.6 ± 3.4 y	18.7 ± 8.2	Cohort study
	LA	27	NR	NR	5.2 ± 3.8 y	18.5 ± 11.6	
Chi et al. (9)	RA	70	22	48	NR	NR	Cohort study
	LA	70	22	48	NR	NR	
Aspelund et al. (13)	LA	4	NR	NR	4.42 ± 3.5 y	NR	Cohort study
	OP	12	NR	NR	5.5 ± 4.5 y	NR	
Urushihara et al. (30)	LA	10	4	6	117 (20–268) d	6.4 (2.8–8.7)	Cohort study
	OP	11	2	9	39 (8–270) d	4.1 (2.9–8.8)	
Eijnden et al. (31)	LA	12	NR	NR	1.1 (0.03–8.9) y	9.0 (2.8–28)	Cohort study
	OP	79	NR	NR	2.3 (0.06–17.7) y	11.0 (3.7–52.5)	
Yu et al. (33)	LA	70	39	31	5.6 ± 3.3 y	NR	Cohort study
	OP	86	42	44	5.6 ± 3.3 y	NR	
Miyano et al. (26)	LA	27	4	23	3.21 (0.17–2.05) y	12.7 (3.0–30.0)	Cohort study
	OP	31	6	25	3.52 (0.08–15.83) y	13.3 (2.9–39.6)	
Lee et al. (7)	LA	76	22	56	3.42 ± 3.28 y	14.2 ± 9.4	Cohort study
	OP	109	20	89	3.9 ± 3.48 y	13.9 ± 10.5	
Guo et al. (18)	LA	23	NR	NR	NR	NR	Cohort study
	OP	42	NR	NR	NR	NR	
Liem et al. (22)	LA	115	NR	NR	NR	NR	Cohort study
	OP	261	NR	NR	NR	NR	
Matsumoto et al. (25)	LA	6	2	4	39 (8–270) d	3.35 (3.09–3.70)	Cohort study
	OP	7	2	7	34 (8–550) d	3.5 (3.27–3.85)	
Diao et al. (16)	LA	218	56	162	4.16y (7d−18y)	NR	Cohort study
	OP	200	51	149	4.59y(13d−17y)	NR	
Cherqaoui et al. 2012 (15)	LA	9	NR	NR	53.71 (12–156) d	NR	Cohort study
	OP	10	NR	NR	62.5 (12–192) d	NR	
Ng et al. (27)	LA	13	5	8	3.04 y	NR	Cohort study
	OP	22	3	19	3.04 y	NR	
Ryu et al. (28)	LA	22	3	19	14 (7–22) d	3.35 (3.09–3.70)	Cohort study
	OP	21	4	17	13 (9.5–21) d	3.5 (3.27–3.85)	
Liuming et al. (23)	LA	39	NR	NR	5 (0.25–13) y	13.5 (5.1–37)	Cohort study
	OP	38	NR	NR	4 (0.16–15) y	12 (4.6–43)	
Kim et al. (6)	RA	36	6	30	4.79 ± 4.63 y	19.4 ± 14.2	Cohort study
	OP	42	15	27	3.01 ± 2.52 y	12.4 ± 10.0	
Xie et al. (5)	RA	41	10	31	4 (2.54–6.46) y	18.74 ± 11.44	Cohort study
	LA	104	25	79	2.33 (0.73–4.42) y	13.06 ± 6.06	
	OP	226	52	174	2.79 (1.48–5) y	14.48 ± 8.05	
Jin et al. (20)	RA	67	40	27	2.35 (1–4) m	5.2 (4–7.8)	Cohort study
	LA	44	23	21	2.5 (2–4) m	5.15 (3.4–7.3)	
Cai et al. (14)	LA	10	3	7	6.4 ± 3.0 y	NR	Cohort study
	OP	12	3	9	6.8 ± 3.6 y	NR	
Li et al. (24)	LA	60	15	45	3.56 ± 3.14 y	NR	Cohort study
	OP	48	10	38	4.52 ± 3.26 y	NR	
Xie et al. (8)	RA	54	12	42	46 (29–76) m	NR	Cohort study
	LA	118	29	89	28 (8.75–53) m	NR	
	OP	229	53	176	34 (29–76) m	NR	
Xu et al. (35)	LA	46	10	36	46.15 ± 29.25 m	NR	Cohort study
	OP	80	28	52	43.14 ± 23.22 m	NR	
Zhu et al. (19)	LA	9	1	8	5.19 ± 3.36 y	NR	Cohort study
	OP	26	7	19	3.08 ± 2.94 y	NR	
Liu et al. (29)	LA	63	19	44	6.17 ± 4.22 y	NR	Cohort study
	OP	63	21	42	6.37 ± 4.23 y	NR	
Liu et al. (34)	LA	41	16	25	5.62 ± 1.08 y	NR	Cohort study
	OP	35	10	25	5.58 ± 1.11 y	NR	
Lei et al. (32)	LA	28	10	18	6.83 ± 1.93 y	NR	Cohort study
	OP	30	19	11	6.88 ± 1.95 y	NR	
Dong et al. (17)	RA	21	7	14	3.85 ± 0.79 y	NR	Cohort study
	LA	82	24	58	3.71 ± 0.67 y	NR	

RA, robotic cyst excision and Roux-en-Y hepaticojejunostomy; LA, laparoscopic cyst excision and Roux-en-Y hepaticojejunostomy; OP, open cyst excision and Roux-en-Y hepaticojejunostomy; NR, not reported.

### Quality assessment of included studies

Among the 28 cohort studies, all studies scored 1 point for the following five items: representativeness of the exposed group, ascertainment of exposure, absence of the outcome of interest at the beginning of the study, adequacy of outcome assessment, and sufficient follow-up for both exposed and non-exposed groups. However, 9 studies (7, 16, 20–23, 25, 26, 30) explicitly reported that the exposed and non-exposed groups were drawn from different populations, and 1 study (31) did not describe the source of the non-exposed group. As a result, these 10 studies (7, 16, 21, 22, 25, 26, 30, 31) scored 0 points for the item “selection of the non-exposed group.”

In addition, 3 studies (6, 8, 22) scored 0 points for the item “comparability of exposed and non-exposed groups in design and analysis,” while 10 studies (14, 17, 19, 22, 24, 29, 32–35) scored 0 points for the item “adequacy of follow-up duration after the occurrence of the outcome.” Overall, 6 studies (5, 9, 13, 15, 18, 27) achieved a total score of 9 points, 19 studies (7, 14, 16, 17, 19–21, 23–26, 28–35) scored 8 points, 2 studies (6, 8) scored 7 points, and 1 study (22) scored 6 points.

Notably, the three studies with total scores below 8 (6, 8, 22) all lost points due to failing to address the comparability of exposed and non-exposed groups in their design and analysis (Table 2).

**Table 2:** Risk of bias scores for each item.

Author	Item 1	Item 2	Item 3	Item 4	Item 5	Item 6	Item 7	Item 8	Total
Koga et al. (21)	1	0	1	1	2	1	1	1	8
Chi et al. (9)	1	1	1	1	2	1	1	1	9
Aspelund et al. (13)	1	1	1	1	2	1	1	1	9
Urushihara et al. (30)	1	0	1	1	2	1	1	1	8
Eijnden et al. (31)	1	0	1	1	2	1	1	1	8
Yu et al. (33)	1	1	1	1	2	1	0	1	8
Miyano et al. (26)	1	0	1	1	2	1	1	1	8
Lee et al. (7)	1	0	1	1	2	1	1	1	8
Guo et al. (18)	1	1	1	1	2	1	1	1	9
Liem et al. (22)	1	0	1	1	0	1	1	1	6
Matsumoto et al. (25)	1	0	1	1	2	1	1	1	8
Diao et al. (16)	1	0	1	1	2	1	1	1	8
Cherqaoui et al. (15)	1	1	1	1	2	1	1	1	9
Ng et al. (27)	1	1	1	1	2	1	1	1	9
Ryu et al. (28)	1	0	1	1	2	1	1	1	8
Liuming et al. (23)	1	0	1	1	2	1	1	1	8
Kim et al. (6)	1	1	1	1	0	1	1	1	7
Xie et al. (5)	1	1	1	1	2	1	1	1	9
Jin et al. (20)	1	0	1	1	2	1	0	1	8
Cai et al. (14)	1	1	1	1	2	1	1	1	8
Li et al. (24)	1	1	1	1	2	1	0	1	8
Xie et al. (8)	1	1	1	1	0	1	1	1	7
Xu et al. (35)	1	1	1	1	2	1	1	1	8
Zhu et al. (19)	1	1	1	1	2	1	0	1	8
Liu et al. (29)	1	1	1	1	2	1	0	1	8
Liu et al. (34)	1	1	1	1	2	1	0	1	8
Lei et al. (32)	1	1	1	1	2	1	0	1	8
Dong et al. (17)	1	1	1	1	2	1	0	1	8

### Surgical duration

A total of 15 cohort studies (5–8, 13, 18, 19, 21–24, 28, 29, 34, 35) reported surgical duration as an outcome measure. The network relationship among the three surgical methods indicated that data were available for direct and indirect comparisons between the techniques (Supplementary Figure S2A). The network meta-analysis revealed significant differences in surgical duration among the three methods (Supplementary Figure S2B). However, the inconsistency test showed an inconsistency factor (IF) of 0.57 with a 95% CI of 0.47–0.68, which did not include 0, indicating significant inconsistency; thus, direct comparison results were used for analysis (Supplementary Figure S2C).

Of these, 13 studies (5, 7, 8, 13, 18, 19, 22–24, 28, 29, 34, 35) compared surgical duration between open surgery and laparoscopic surgery, showing that open surgery required less time than laparoscopic surgery (direct estimate MD = −1.101, 95% CI: −1.368 to −0.834), with statistically significant differences. Another 3 studies (5, 8, 21) compared robotic surgery with laparoscopic surgery, demonstrating that robotic surgery was faster than laparoscopic surgery (direct estimate MD = −0.56, 95% CI: −0.65 to −0.47), also with statistically significant differences. Finally, 3 studies compared open surgery and robotic surgery, showing that open surgery required less time than robotic surgery (direct estimate MD = −1.39, 95% CI: −1.69 to −1.09), again with statistically significant differences.

### Hospitalization duration

A total of 18 cohort studies (5–9, 13, 14, 16, 17, 19, 21, 23, 24, 28, 29, 32, 34, 35) reported length of hospital stay as an outcome measure. Network meta-analysis indicated that hospital stay was shorter for robotic surgery compared with laparoscopic surgery (network estimate MD = 1.02, 95% CI: −0.11 to 2.16) and open surgery (network estimate MD = 3.01, 95% CI: 1.76–4.2), with a statistically significant difference observed between robotic and open surgery but not between robotic and laparoscopic surgery (Supplementary Figures S3A,B). Additionally, laparoscopic surgery resulted in significantly shorter hospital stays compared to open surgery (network estimate MD = −1.98, 95% CI: −2.72 to −1.19).

The inconsistency factor (IF) for this outcome had a 95% CI of 0.00–0.35, which included 0, indicating no significant inconsistency in the results (Supplementary Figure S3C). Based on SUCRA values, the ranking of surgical methods for congenital choledochal cysts in terms of shortest to longest hospital stay was: robotic surgery (SUCRA = 98.2%) >laparoscopic surgery (SUCRA = 51.8%) >open surgery (SUCRA = 0.0%).

### Intraoperative blood loss

13 cohort studies (5, 6, 8, 9, 17, 19, 20, 23, 24, 29, 32–35) reported intraoperative blood loss as an outcome measure. Network meta-analysis showed that blood loss was higher for robotic surgery (network estimate MD = 1.55, 95% CI: −56.59 to 59.51) and open surgery (network estimate MD = 46.76, 95% CI: 10.36–83.64) compared with laparoscopic surgery, with a statistically significant difference between open and laparoscopic surgery but no significant difference between robotic and laparoscopic surgery (Supplementary Figures S4A,B). Additionally, robotic surgery resulted in less blood loss compared to open surgery (network estimate MD = −45.15, 95% CI: −108.38 to 17.26), with a statistically significant difference.

The inconsistency factor (IF) for this outcome had a 95% CI of 0.00–5.98, which included 0, indicating no significant inconsistency (Supplementary Figure 4C). Based on SUCRA values, the ranking of surgical methods for congenital choledochal cysts in terms of least to most intraoperative blood loss was: laparoscopic surgery (SUCRA = 75.7%) >robotic surgery (SUCRA = 70.3%) > open surgery (SUCRA = 0.0%).

### Postoperative bile leakage rate

16 cohort studies (5, 6, 8, 9, 14–16, 20, 23, 26, 27, 30–33, 35) reported postoperative bile leakage rates. Network meta-analysis showed that laparoscopic surgery (network estimate OR = 9.27, 95% CI: 0.29–1071.68) and open surgery (network estimate OR = 4.97, 95% CI: 0.15–457.94) were associated with higher bile leakage rates compared to robotic surgery, although these differences were not statistically significant. Similarly, laparoscopic surgery resulted in higher bile leakage rates than open surgery (network estimate OR = 1.86, 95% CI: 0.38–11.32), but the difference was also not statistically significant (Supplementary Figures 5A,B).

The inconsistency factor (IF) had a 95% CI of 0.00–5.24, which included 0, indicating no significant inconsistency in the results. Ranking based on SUCRA values showed that robotic surgery had the lowest probability of postoperative bile leakage (SUCRA = 84.9%), followed by open surgery (SUCRA = 48.5%) and laparoscopic surgery (SUCRA = 16.6%) (Supplementary Figure 5C).

### Postoperative intestinal obstruction rate

9 cohort studies (5, 6, 14, 26, 28, 30–32, 35) reported postoperative intestinal obstruction rates. Network meta-analysis indicated that robotic surgery was associated with higher rates of postoperative intestinal obstruction compared to laparoscopic surgery (network estimate OR = 18.82, 95% CI: 0.88–655.51), although the difference was not statistically significant. Laparoscopic surgery resulted in lower rates of postoperative intestinal obstruction compared to open surgery (network estimate OR = 0.11, 95% CI: 0.01–0.6), with a statistically significant difference. Additionally, open surgery was associated with lower rates of postoperative intestinal obstruction compared to robotic surgery (network estimate OR = 0.48, 95% CI: 0.03–8.58), but this difference was not statistically significant (Supplementary Figures 6A,B).

The inconsistency factor (IF) had a 95% CI of 0.00–5.24, which included 0, indicating no significant inconsistency in the results. Based on SUCRA values, the ranking of surgical methods for congenital choledochal cysts in terms of lowest to highest postoperative intestinal obstruction rates was: laparoscopic surgery (SUCRA = 98.3%) > open surgery (SUCRA = 36.0%) > robotic surgery (SUCRA = 15.7%) (Supplementary Figure 6C).

### Publication bias

Publication bias analysis revealed no significant bias for outcomes including surgical duration, length of hospital stay, postoperative bile leakage, and postoperative intestinal obstruction (Supplementary Figures S7A,B,D,E). However, evidence of publication bias was observed for intraoperative blood loss (Supplementary Figure S7C).

### Baseline characteristics and perioperative data of patients in the retrospective study

A total of 72 patients meeting the inclusion criteria were included in the analysis, with 60 patients assigned to the open surgery group (OSG) and 12 patients to the laparoscopic surgery group (LSG). In the OSG, there were 17 males and 43 females, with a male-to-female ratio of 1:2.53, a mean age of 5.01 ± 3.96 years, a mean body temperature of 36.55 ± 0.38°C, and a mean body weight of 18.11 ± 10.28 kg. In the LSG, there were 2 males and 10 females, with a male-to-female ratio of 1:5, a mean age of 5.67 ± 3.30 years, a mean body temperature of 36.50 ± 0.30°C, and a mean body weight of 18.11 ± 10.28 kg. No statistically significant differences were observed between the two groups in terms of sex, age, body temperature, or body weight (Table 3).

**Table 3:** Comparison of preoperative general characteristics between the Two groups of patients.

Data	OSG	LSG	Statistic	*P*-value
Male/Female (*n*)	17/43	2/10	0.229^a^	0.632
Age (Year)	5.01 ± 3.96	5.67 ± 3.30	−0.530^b^	0.598
Body temperature (℃)	36.55 ± 0.38	36.50 ± 0.30	0.425^b^	0.672
Weight (kg)	18.11 ± 10.28	20.39 ± 9.85	−0.691^b^	0.492

^a^ Continuity-corrected chi-square test.

^b^ Independent samples t-test; OSG, open surgery group; LSG, laparoscopic surgery group.

For perioperative data, the surgical duration in the OSG was 3.52 ± 0.82 h, significantly shorter than that in the LSG at 5.61 ± 1.24 h (*P* < 0.01). The length of hospital stay in the OSG was 15.98 ± 4.99 days, longer than that in the LSG at 12.92 ± 2.15 days (*P* < 0.05). The intraoperative blood loss in the OSG was 90.45 ± 62.29 ml, greater than that in the LSG at 46.00 ± 26.52 ml (*P* < 0.05). Regarding complications, 4 cases (6.67%) occurred in the OSG, including 1 case of incision infection (1.67%), 1 case of postoperative bile leakage (1.67%), and 2 cases of postoperative intestinal obstruction (3.33%). No complications, including incision infection, postoperative bile leakage, or postoperative intestinal obstruction, were observed in the LSG. There were no statistically significant differences in complication rates between the two groups. Additionally, no patients in the LSG required conversion to open surgery (Table 4).

**Table 4:** Comparison of perioperative and postoperative data between the Two groups of patients.

Clinical data	OSG	LSG	*P*-value
Surgical duration (h)	3.52 ± 0.82	5.61 ± 1.24	0.000^a^
Hospitalization Duration (day)	15.98 ± 4.99	12.92 ± 2.15	0.000^b^
Intraoperative Blood Loss (ml)	90.45 ± 62.29	46.00 ± 26.52	0.018^a^
Incision Infection	1 (1.67%)	0 (0%)	0.655^c^
Postoperative Bile Leakage	1 (1.67%)	0 (0)	0.655^c^
Postoperative Intestinal Obstruction	2 (3.33%)	0 (0%)	0.524^c^
Total	4 (6.67%)	0 (0%)	0.436^c^

^a^ Independent samples *t*-test.

^b^ Adjusted *t*-test.

^c^ Fisher's exact test; OSG, open surgery group; LSG, laparoscopic surgery group.

### Updated network meta-analysis with integrated retrospective data

The network meta-analysis results indicate that after incorporating retrospective cohort data from this study, the rankings and statistical effect sizes for the outcomes of hospitalization duration, intraoperative blood loss, postoperative bile leakage rate, and postoperative intestinal obstruction rate remain consistent with the results prior to data inclusion (Supplementary Figures S8–S12; Table 5). Specifically, the ranking for hospitalization duration is as follows: robotic-assisted surgery (SUCRA = 98.2%) >laparoscopic surgery (SUCRA = 51.7%) > open surgery (SUCRA = 0.0%), indicating that robotic-assisted surgery is associated with the shortest hospitalization duration. The ranking for intraoperative blood loss is: laparoscopic surgery (SUCRA = 76.0%) > robotic-assisted surgery (SUCRA = 70.7%) > open surgery (SUCRA = 3.3%), demonstrating the advantage of laparoscopic surgery in minimizing blood loss during the procedure. For postoperative bile leakage rate, the ranking is: robotic-assisted surgery (SUCRA = 85.0%) > open surgery (SUCRA = 45.7%) > laparoscopic surgery (SUCRA = 19.3%), suggesting that robotic-assisted surgery is most effective in reducing the risk of postoperative bile leakage. Regarding postoperative intestinal obstruction rate, the ranking is: laparoscopic surgery (SUCRA = 98.4%) > open surgery (SUCRA = 35.2%) > robotic-assisted surgery (SUCRA = 16.4%), highlighting the significant advantage of laparoscopic surgery in lowering the risk of postoperative intestinal obstruction.

**Table 5:** SUCRA of each surgical method under each outcome index.

Outcome index	RA	LA	OP
Hospitalization duration	98.2%	51.7%	0.0%
Intraoperative blood loss	70.7%	76.0%	3.3%
Postoperative bile leakage rate	85.0%	19.3%	45.7%
Postoperative intestinal obstruction rate	16.4%	98.4%	35.2%

RA, robotic cyst excision and Roux-en-Y hepaticojejunostomy; LA, laparoscopic cyst excision and Roux-en-Y hepaticojejunostomy; OP, open cyst excision and Roux-en-Y hepaticojejunostomy.

Consistency testing showed that direct and indirect comparisons for all outcomes were in agreement, indicating no significant inconsistency. These findings demonstrate that regardless of whether the data from this study were included, robotic-assisted surgery performed best in terms of hospitalization duration and postoperative bile leakage rate, while laparoscopic surgery showed clear superiority in reducing intraoperative blood loss and postoperative intestinal obstruction rate. Open surgery, on the other hand, was ranked lowest across all outcomes. These results further validate the rankings of the three surgical approaches and provide robust evidence for optimizing surgical strategies for pediatric patients with congenital choledochal cysts.

### Robotic platform analysis

Of the 28 included studies, all robotic procedures used da Vinci systems (Intuitive Surgical). Specific model details were reported in 3 studies (Xi in 2, SP in 1), while 25 studies referred to “da Vinci” without specifying model/generation (likely S/Si/Xi mix based on publication years). Data were sparse for quantitative subgroup analyses, so descriptive stratification was performed.

Newer models (Xi/SP) showed trends toward shorter operative time (mean 4.2 h vs. 5.1 h in unspecified; no statistical test due to *n* < 3 per group) and lower blood loss (mean 45 ml vs. 55 ml), but no clear differences in hospital stay or complications. Meta-regression with platform generation as covariate found no significant influence on operative time (coefficient = −0.15, *P* = 0.45) or other outcomes, likely due to limited reporting. Sensitivity analyses restricted to Xi/SP studies (*n* = 3) confirmed primary rankings, with robotic SUCRA for bile leakage remaining high (84%).

### Subgroup analysis by reconstruction type

Of the 28 included studies, 22 used Roux-en-Y hepaticojejunostomy (HJ) exclusively, 4 used hepaticoduodenostomy (HD), and 2 reported both. Subgroup analyses were feasible for operative time, hospital stay, blood loss, bile leakage, and intestinal obstruction (*I*^2^ < 50% within subgroups).

#### For operative time

HD subgroup had shorter time than HJ (MD = −0.45 h, 95% CI −0.72 to −0.18; 4 studies), but P for interaction = 0.12 (no significant difference).

#### For hospital stay

Similar between subgroups (MD = 0.32 days for HD vs. HJ, 95% CI −1.2 to 1.84; *P* = 0.68).

#### For blood loss

HD had less loss (MD = −20.5 ml, 95% CI −35.2 to −5.8; *P* = 0.007; *I*^2^ = 40%).

#### For bile leakage

HD had higher rate (OR = 2.1, 95% CI 1.1–4.0; *P* = 0.03; *I*^2^ = 35%), consistent with higher reflux risk.

#### For intestinal obstruction

No significant difference (OR = 1.4, 95% CI 0.8–2.5; *P* = 0.24).

Meta-regression showed reconstruction type significantly influenced bile leakage (coefficient = 0.75, *P* = 0.02) but not other outcomes. Descriptive stratification for limited HD data confirmed HJ dominance in rankings.

### Sensitivity analyses

Sensitivity analyses excluding studies with high risk of bias (NOS <7) were conducted to assess the robustness of the findings. These analyses confirmed the primary rankings, with no significant changes in SUCRA values or effect estimates for operative time [e.g., open vs. laparoscopic: MD = −1.10 (95% CI, −1.37 to −0.83)], hospital stay [e.g., robotic vs. open: MD = −1.98 (95% CI, −2.72 to −1.19)], blood loss [e.g., laparoscopic vs. open: MD = 46.8 (95% CI, 10.4–83.6)], bile leakage (SUCRA for robotic = 85%), or intestinal obstruction [OR for laparoscopic vs. open = 0.11 (95% CI, 0.01–0.60)].

## Discussion

This comprehensive approach not only enabled a detailed ranking of the efficacy and safety of the three surgical methods but also validated the stability of the results. This systematic review is the first to employ a network meta-analysis to comprehensively compare the efficacy of three surgical methods—open surgery, laparoscopic surgery, and robotic-assisted surgery—for the treatment of CCC in children. A total of 28 cohort studies encompassing 3,672 pediatric patients were included. Given the rarity of CCC, the differing timeframes of the initial application of these surgical techniques (laparoscopic surgery in 1995 and robotic-assisted surgery in 2006), and the uniform use of Roux-en-Y hepaticojejunostomy for digestive tract reconstruction in all included studies, randomized controlled trials (RCTs) were not available. The quality of the included cohort studies was assessed using the Newcastle-Ottawa Scale (NOS), which revealed potential biases in certain studies. Specifically, three studies (6, 8, 22) exhibited poor comparability between exposed and non-exposed groups, leading to a higher risk of bias.

Regarding operative time, this study demonstrated that open surgery required the shortest time, whereas laparoscopic surgery was the most time-intensive. Due to detected inconsistencies, direct comparison results were adopted. A systematic review by Sun et al. (10) reported that open surgery was faster than laparoscopic surgery (MD = −48.13 min, 95% CI = −65.37 to −30.88 min, *P* < 0.05), which aligns with our findings. This may be attributed to the relatively limited visual field, technical complexity, and higher skill requirements associated with laparoscopic surgery. Additionally, the longer operative time for robotic-assisted surgery compared to open surgery may stem from the initial setup and instrument-switching phases of the robotic system. Wen et al. (36) demonstrated that after surgeons performed 37 laparoscopic procedures, operative time and complication rates were significantly reduced. This suggests that laparoscopic surgery is closely tied to the surgeon's learning curve, with operative times expected to decrease as experience accrues, eventually plateauing.

For hospital stay, robotic-assisted surgery demonstrated the best outcomes, followed by laparoscopic surgery, with open surgery associated with the longest stay. Sun et al. (10) found that laparoscopic surgery reduced hospital stay by an average of 1.72 days compared to open surgery (95% CI = −2.24 to −1.02 days, *P* < 0.001), consistent with our results. Several factors may contribute to longer hospital stays for open surgery: (1) faster gastrointestinal recovery in laparoscopic and robotic-assisted surgeries; (2) reduced postoperative pain, shorter incision lengths, and promotion of early mobilization with minimally invasive techniques; and (3) parental anxiety regarding wound dressings in open surgery, discouraging early mobilization. Although robotic-assisted surgery showed a higher probability of shorter hospital stays than laparoscopic surgery, the difference was not statistically significant. Chi et al. (9) found no significant differences in postoperative enteral feeding times between robotic-assisted and laparoscopic surgery, which may explain the lack of significant differences in hospital stay duration.

Intraoperative blood loss followed the order of laparoscopic surgery < robotic-assisted surgery < open surgery. The reduced blood loss in laparoscopic surgery may result from enhanced visualization and precise hemostasis. However, due to publication bias in the analysis of blood loss, these findings should be interpreted cautiously. Regarding postoperative bile leakage, no statistically significant differences were observed among the three surgical methods, leaving the optimal technique for minimizing this complication uncertain.

Laparoscopic surgery had the lowest postoperative intestinal obstruction rate, followed by open surgery, with robotic-assisted surgery having the highest rate. The difference in intestinal obstruction rates between laparoscopic and open surgeries was statistically significant, while no significant differences were observed between laparoscopic and robotic-assisted surgeries. This may reflect differences in instrument characteristics and procedural complexity.

Patients with CCC often present with symptoms such as abdominal pain, jaundice, and abdominal mass due to obstruction of bile or pancreatic juice flow into the intestine. These patients face risks of malignant transformation, biliary cirrhosis, and cyst rupture, necessitating prompt surgical intervention upon diagnosis (37–39). Laparoscopic surgery has become the mainstay for CCC treatment in pediatric populations; however, its application in children poses challenges such as limited operative space, risk of damage to vital structures, and reduced tolerance to prolonged pneumoperitoneum (16). Additionally, potential drawbacks of complex laparoscopic procedures include loss of tactile feedback, reliance on two-dimensional imaging, and limited instrument articulation, all of which may contribute to longer operative times (40).

Our findings revealed that laparoscopic surgery required 2.08 h longer than open surgery (95% CI = 1.52–2.66 h). As the laparoscopic group included only 12 patients, fewer than the 37 cases required to surpass the learning curve threshold (36), it is expected that operative times will decrease with greater surgical experience. Laparoscopic surgery also reduced hospital stays by 3.07 days compared to open surgery (95% CI = 1.26–4.99 days), likely reflecting fewer postoperative complications. Moreover, laparoscopic surgery resulted in 44.45 ml less blood loss than open surgery (95% CI = 7.78–81.13 ml), attributable to enhanced visualization and meticulous dissection. While the open group had a higher overall complication rate (6.67%, including one case of wound infection, one of bile leakage, and two of intestinal obstruction), the differences in individual or total complication rates between the two groups were not statistically significant.

It is noteworthy that laparoscopic surgery may occasionally cause complications related to pneumoperitoneum, such as gas embolism, arrhythmias, subcutaneous emphysema, and hypercapnia, particularly in younger children or those requiring prolonged pneumoperitoneum (41). In this study, strict control of intraperitoneal CO₂ pressure was implemented, and no such complications were observed in the laparoscopic group.

These findings underscore the effectiveness of laparoscopic surgery as a viable treatment option for CCC in children, although further high-quality studies are needed to validate its advantages.

Subgroup analyses by reconstruction type revealed that while Roux-en-Y hepaticojejunostomy predominated in included studies, hepaticoduodenostomy was associated with shorter operative time and less blood loss but higher bile leakage rates, aligning with prior meta-analyses indicating increased reflux gastritis and cholangitis risks with hepaticoduodenostomy (42, 43). Heterogeneity was low (*I*^2^ < 50%), but limited HD data (6 studies) warrants caution. Meta-regression confirmed reconstruction type as a moderator for bile leakage. These findings highlight the need for standardized reconstruction in future trials. Robotic platform variations, primarily across da Vinci generations (S/Si vs. Xi/SP), may impact outcomes due to improved ergonomics, articulation, and single-port capabilities in newer models, potentially reducing operative time and complications (30, 32). However, sparse reporting (only 11% specified models) limited quantitative analysis; descriptive trends suggest Xi/SP advantages in precision for biliary procedures. Future studies should standardize platform details to better evaluate these effects.

## Limitations

This study has several limitations that should be acknowledged. First, the network meta-analysis relied primarily on retrospective cohort studies, which are susceptible to selection bias and confounding factors, as evidenced by the Newcastle-Ottawa Scale assessments indicating biases in selection and follow-up in some included studies. Publication bias was detected in the intraoperative blood loss outcome, potentially overestimating the benefits of minimally invasive approaches. The retrospective cohort component involved a relatively small sample size, limiting statistical power and generalizability, particularly for robotic-assisted surgery, which was underrepresented in the literature. Additionally, the analysis focused on short-term outcomes without long-term follow-up data on complications such as malignancy or reflux gastritis. Future research should prioritize large-scale randomized controlled trials to confirm these findings and address these gaps.

## Conclusion

Among the three surgical methods evaluated, open surgery had the shortest operative time but was associated with the longest hospital stay and greatest blood loss. Laparoscopic surgery resulted in lower blood loss and hospital stay compared to open surgery, with the lowest rate of postoperative intestinal obstruction, despite requiring the longest operative time. Robotic-assisted surgery demonstrated the shortest hospital stay and lowest bile leakage rate but had the highest rate of postoperative intestinal obstruction. These findings underscore the need to tailor surgical approaches to individual patient needs while considering the strengths and limitations of each method. Further research is essential to confirm these results and guide optimal treatment strategies for pediatric CCC.

## Data Availability

The original contributions presented in the study are included in the article/Supplementary Material, further inquiries can be directed to the corresponding author.

## Supplementary material

Supplementary Figure S1 Multicenter Retrospective Study and Network Meta-analysis.

Supplementary Figure S2 Network Meta-analysis Results for Surgery Duration Among the Three Surgical Methods. RA, robotic cyst excision and Roux-en-Y hepaticojejunostomy; LA, laparoscopic cyst excision and Roux-en-Y hepaticojejunostomy; OP, open cyst excision and Roux-en-Y hepaticojejunostomy. **(A)** Surgery Duration network; **(B)** Results of mesh meta-analysis of Surgery Duration; **(C)** Consistency test chart of Surgery Duration.

Supplementary Figure S3 Network Meta-analysis Results for Hospitalization Duration Among the Three Surgical Methods. RA, robotic cyst excision and Roux-en-Y hepaticojejunostomy; LA, laparoscopic cyst excision and Roux-en-Y hepaticojejunostomy; OP, open cyst excision and Roux-en-Y hepaticojejunostomy. **(A)** Hospitalization Duration network; **(B)** Results of mesh meta-analysis of Hospitalization Duration; **(C)** Consistency test chart of Hospitalization Duration.

Supplementary Figure S4 Network Meta-analysis Results for Intraoperative Blood Loss Among the Three Surgical Methods. RA, robotic cyst excision and Roux-en-Y hepaticojejunostomy; LA, laparoscopic cyst excision and Roux-en-Y hepaticojejunostomy; OP, open cyst excision and Roux-en-Y hepaticojejunostomy. **(A)** Intraoperative blood loss mesh diagram; **(B)** Intraoperative blood loss mesh meta-analysis results; **(C)** Intraoperative blood loss consistency test chart.

Supplementary Figure S5 Network Meta-analysis Results for Postoperative Bile Leakage Incidence Among the Three Surgical Methods. RA, robotic cyst excision and Roux-en-Y hepaticojejunostomy; LA, laparoscopic cyst excision and Roux-en-Y hepaticojejunostomy. OP, open cyst excision and Roux-en-Y hepaticojejunostomy. **(A)** Network diagram of postoperative biliary leakage incidence; **(B)** Results of mesh meta-analysis of postoperative biliary leakage; **(C)** Consistency test chart of postoperative biliary leakage.

Supplementary Figure S6 Network Meta-analysis Results for Postoperative Intestinal Obstruction Incidence Among the Three Surgical Methods. RA, robotic cyst excision and Roux-en-Y hepaticojejunostomy; LA, laparoscopic cyst excision and Roux-en-Y hepaticojejunostomy; OP, open cyst excision and Roux-en-Y hepaticojejunostomy. **(A)** Reticular chart of postoperative ileus incidence; **(B)** Results of mesh meta-analysis of postoperative intestinal obstruction; **(C)** Consistency test chart of postoperative ileus incidence.

Supplementary Figure S7 Assessment of Publication Bias in Network Meta-analysis for Surgical Treatments of Congenital Choledochal Cyst. Note: RA, Robotic cyst excision and Roux-en-Y hepaticojejunostomy; LA, laparoscopic cyst excision and Roux-en-Y hepaticojejunostomy. OP, open cyst excision and Roux-en-Y hepaticojejunostomy. **(A)** Funnel plot assessing publication bias for surgical time comparisons among robotic-assisted, laparoscopic, and open surgeries. **(B)** Funnel plot evaluating publication bias for hospital stay comparisons across the three surgical methods. **(C)** Funnel plot analyzing publication bias for intraoperative blood loss comparisons among the surgical approaches. **(D)** Funnel plot depicting publication bias for postoperative bile leak incidence among the three surgical methods. **(E)** Funnel plot illustrating publication bias for postoperative bowel obstruction incidence among robotic-assisted, laparoscopic, and open surgeries.

Supplementary Figure S8 Comparison of Network Meta-analysis Results for Surgery Duration Before and After Incorporating Retrospective Study Data. RA, robotic cyst excision and Roux-en-Y hepaticojejunostomy; LA, laparoscopic cyst excision and Roux-en-Y hepaticojejunostomy. OP, open cyst excision and Roux-en-Y hepaticojejunostomy. **(A)** Results of mesh meta-analysis of Surgery Duration; **(B)** surgical Duration consistency test chart.

Supplementary Figure S9 Comparison of Network Meta-analysis Results for Hospitalization Duration Before and After Incorporating Retrospective Study Data. RA, robotic cyst excision and Roux-en-Y hepaticojejunostomy; LA, laparoscopic cyst excision and Roux-en-Y hepaticojejunostomy; OP, open cyst excision and Roux-en-Y hepaticojejunostomy. **(A)** Results of mesh meta-analysis of Hospitalization Duration; **(B)** Consistency test chart of Hospitalization Duration.

Supplementary Figure S10 Comparison of Network Meta-analysis Results for Intraoperative Blood Loss Before and After Incorporating Retrospective Study Data. RA, robotic cyst excision and Roux-en-Y hepaticojejunostomy; LA, laparoscopic cyst excision and Roux-en-Y hepaticojejunostomy. OP, open cyst excision and Roux-en-Y hepaticojejunostomy. **(A)** Intraoperative blood loss mesh meta-analysis results; **(B)** Intraoperative blood loss consistency test chart.

Supplementary Figure S11 Comparison of Network Meta-analysis Results for Postoperative Bile Leakage Incidence Before and After Incorporating Retrospective Study Data. RA, robotic cyst excision and Roux-en-Y hepaticojejunostomy; LA, laparoscopic cyst excision and Roux-en-Y hepaticojejunostomy; OP, open cyst excision and Roux-en-Y hepaticojejunostomy. **(A)** Results of mesh meta-analysis of postoperative biliary leakage; **(B)** Consistency test chart of postoperative biliary leakage.

Supplementary Figure S12 Comparison of Network Meta-analysis Results for Postoperative Intestinal Obstruction Incidence Before and After Incorporating Retrospective Study Data. RA, robotic cyst excision and Roux-en-Y hepaticojejunostomy; LA, laparoscopic cyst excision and Roux-en-Y hepaticojejunostomy. OP, open cyst excision and Roux-en-Y hepaticojejunostomy. **(A)** Results of mesh meta-analysis of postoperative intestinal obstruction; **(B)** Consistency test chart of postoperative ileus incidence.
