# Supplementary material for: Evaluating surgical strategies for pediatric congenital choledochal cysts: a multicenter retrospective study and network meta-analysis
Source: Front Pediatr. 2025 Sep 26;13:1678421. doi: 10.3389/fped.2025.1678421 (PMC12510841; doi:10.3389/fped.2025.1678421)
Supplement: Supplementary Figure S1 — Multicenter Retrospective Study and Network Meta-analysis. [file Datasheet1.docx]

#1 (("Choledochal Cyst"[Mesh]) OR ((((((((((("Choledochal

Cyst"[Title/Abstract]) OR ("Choledochal Cysts"[Title/Abstract])) OR

("Choledochal Cysts"[Title/Abstract])) OR ("Bile Duct

Cyst"[Title/Abstract])) OR (Choledochocele[Title/Abstract])) OR

(Choledochoceles[Title/Abstract])) OR ("Choledochal

Diverticulum"[Title/Abstract])) OR ("Choledochal

Diverticulums"[Title/Abstract])) OR ("Congenital Biliary

Dilatation"[Title/Abstract])) OR ("Congenital Biliary

Dilatations"[Title/Abstract])) OR ("Common Bile Duct Choledochal

Cyst"[Title/Abstract]))) OR (("Caroli Disease"[Mesh]) OR (((((("Caroli

Disease"[Title/Abstract]) OR ("Caroli's Disease"[Title/Abstract])) OR

("Carolis Disease"[Title/Abstract])) OR ("Caroli's

Syndrome"[Title/Abstract])) OR ("Caroli Syndrome"[Title/Abstract])) OR

("Carolis Syndrome"[Title/Abstract])))

#2 ("Laparoscopy"[Mesh]) OR ((((((((((((Laparoscopy[Title/Abstract]) OR

(Laparoscopies[Title/Abstract])) OR (Celioscopy[Title/Abstract])) OR

(Celioscopies[Title/Abstract])) OR (Peritoneoscopy[Title/Abstract])) OR

(Peritoneoscopies[Title/Abstract])) OR ("Laparoscopic Surgical

Procedures"[Title/Abstract])) OR ("Laparoscopic Surgical

Procedure"[Title/Abstract])) OR ("Laparoscopic Surgery"[Title/Abstract]))

OR ("Laparoscopic Surgeries"[Title/Abstract])) OR ("Laparoscopic Assisted

Surgery"[Title/Abstract])) OR ("Laparoscopic Assisted

Surgeries"[Title/Abstract]))

#3 ("Robotic Surgical Procedures"[Mesh]) OR (((((((((((((((("Robotic

Surgical Procedures"[Title/Abstract]) OR ("Robotic Surgical

Procedure"[Title/Abstract])) OR ("Robot Surgery"[Title/Abstract])) OR

("Robot Surgeries"[Title/Abstract])) OR ("Robot-Assisted

Surgery"[Title/Abstract])) OR ("Robot Assisted Surgery"[Title/Abstract]))

OR ("Robot-Assisted Surgeries"[Title/Abstract])) OR ("Robot-Enhanced

Procedures"[Title/Abstract])) OR ("Robot Enhanced

Procedures"[Title/Abstract])) OR ("Robot-Enhanced

Procedure"[Title/Abstract])) OR ("Robotic-Assisted

Surgery"[Title/Abstract])) OR ("Robotic Assisted Surgery"[Title/Abstract]))

OR ("Robotic-Assisted Surgeries"[Title/Abstract])) OR ("Robot-Enhanced

Surgery"[Title/Abstract])) OR ("Robot Enhanced Surgery"[Title/Abstract]))

#4 ("Biliary Tract Surgical Procedures"[Mesh]) OR ((((((((((((("Biliary Tract

Surgical Procedures"[Title/Abstract]) OR ("Biliary Tract Surgical

Procedure"[Title/Abstract])) OR ("Biliary Surgical

Procedure"[Title/Abstract])) OR ("Biliary Surgical

Procedure"[Title/Abstract])) OR ("Biliary Tract Surgery"[Title/Abstract]))

OR ("Bile Duct Operation"[Title/Abstract])) OR ("Bile Duct

Surgery"[Title/Abstract])) OR ("Bile Tract Surgery"[Title/Abstract])) OR

("Biliary Surgery"[Title/Abstract])) OR ("Biliary Tract

Operation"[Title/Abstract])) OR ("Biliary Tract

Reoperation"[Title/Abstract]) ) OR ("Bile Tract

Reconstruction"[Title/Abstract])) OR ("Bile Duct

Reconstruction"[Title/Abstract]))

#5 ("Surgical Procedures, Operative"[Mesh]) OR ((((("Operative

Procedures"[Title/Abstract]) OR ("Operative Procedure"[Title/Abstract]))

OR ("Surgical Procedures"[Title/Abstract])) OR ("Surgical

Procedure"[Title/Abstract])) OR ("Ghost Surgery"[Title/Abstract]))

#6 #2 OR #3 OR #4 OR #5

#7 #1 AND #6
