# Supplementary figures and images for: Evaluating surgical strategies for pediatric congenital choledochal cysts: a multicenter retrospective study and network meta-analysis
Source: Front Pediatr. 2025 Sep 26;13:1678421. doi: 10.3389/fped.2025.1678421 (PMC12510841; doi:10.3389/fped.2025.1678421)

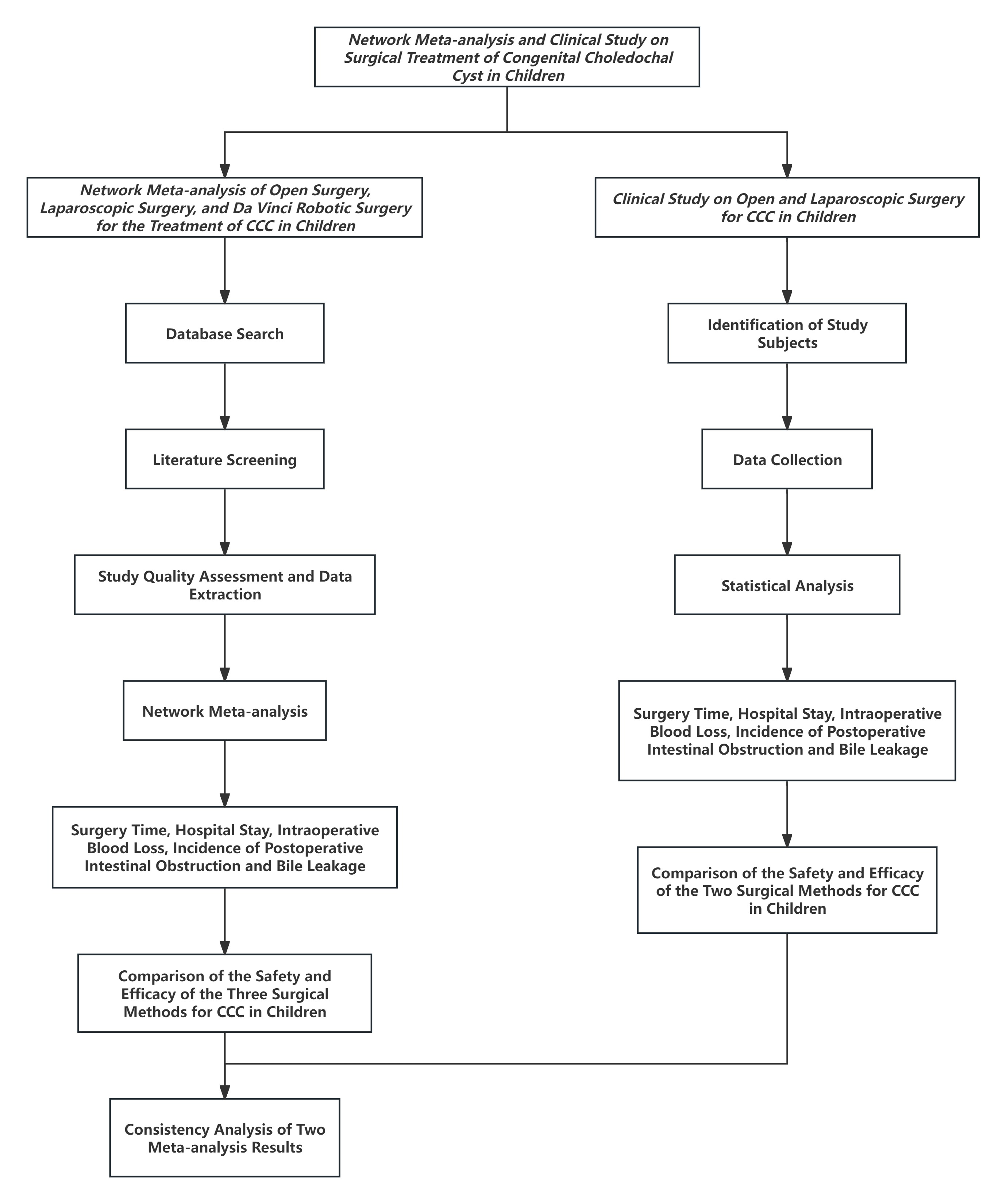

Supplement: Supplementary Figure S2 — Network Meta-analysis Results for Surgery Duration Among the Three Surgical Methods. RA, robotic cyst excision and Roux-en-Y hepaticojejunostomy; LA, laparoscopic cyst excision and Roux-en-Y hepaticojejunostomy; OP, open cyst excision and Roux-en-Y hepaticojejunostomy. (A) Surgery Duration network; (B) Results of mesh meta-analysis of Surgery Duration; (C) Consistency test chart of Surgery Duration. [file Image1.tif]

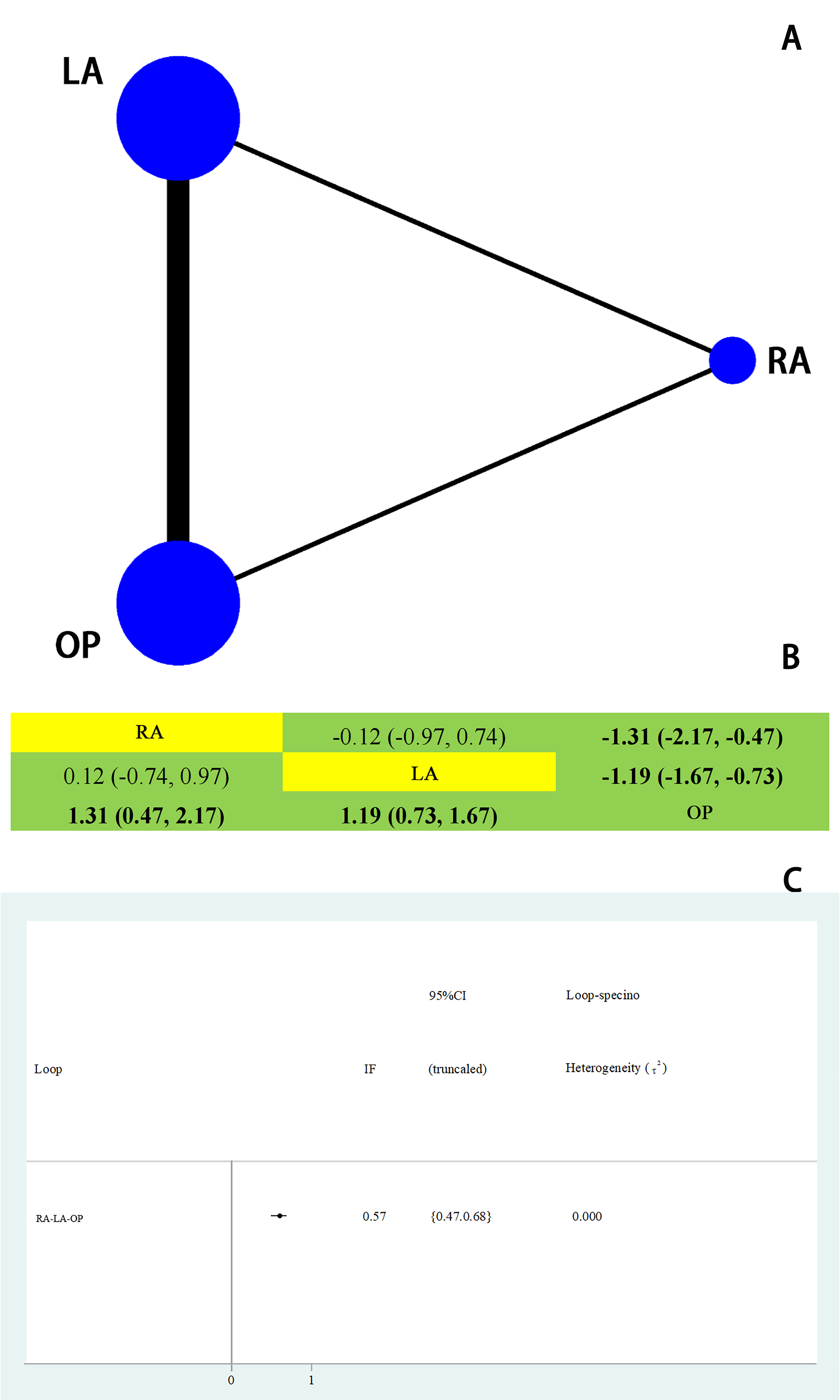

Supplement: Supplementary Figure S3 — Network Meta-analysis Results for Hospitalization Duration Among the Three Surgical Methods. RA, robotic cyst excision and Roux-en-Y hepaticojejunostomy; LA, laparoscopic cyst excision and Roux-en-Y hepaticojejunostomy; OP, open cyst excision and Roux-en-Y hepaticojejunostomy. (A) Hospitalization Duration network; (B) Results of mesh meta-analysis of Hospitalization Duration; (C) Consistency test chart of Hospitalization Duration. [file Image2.jpeg]

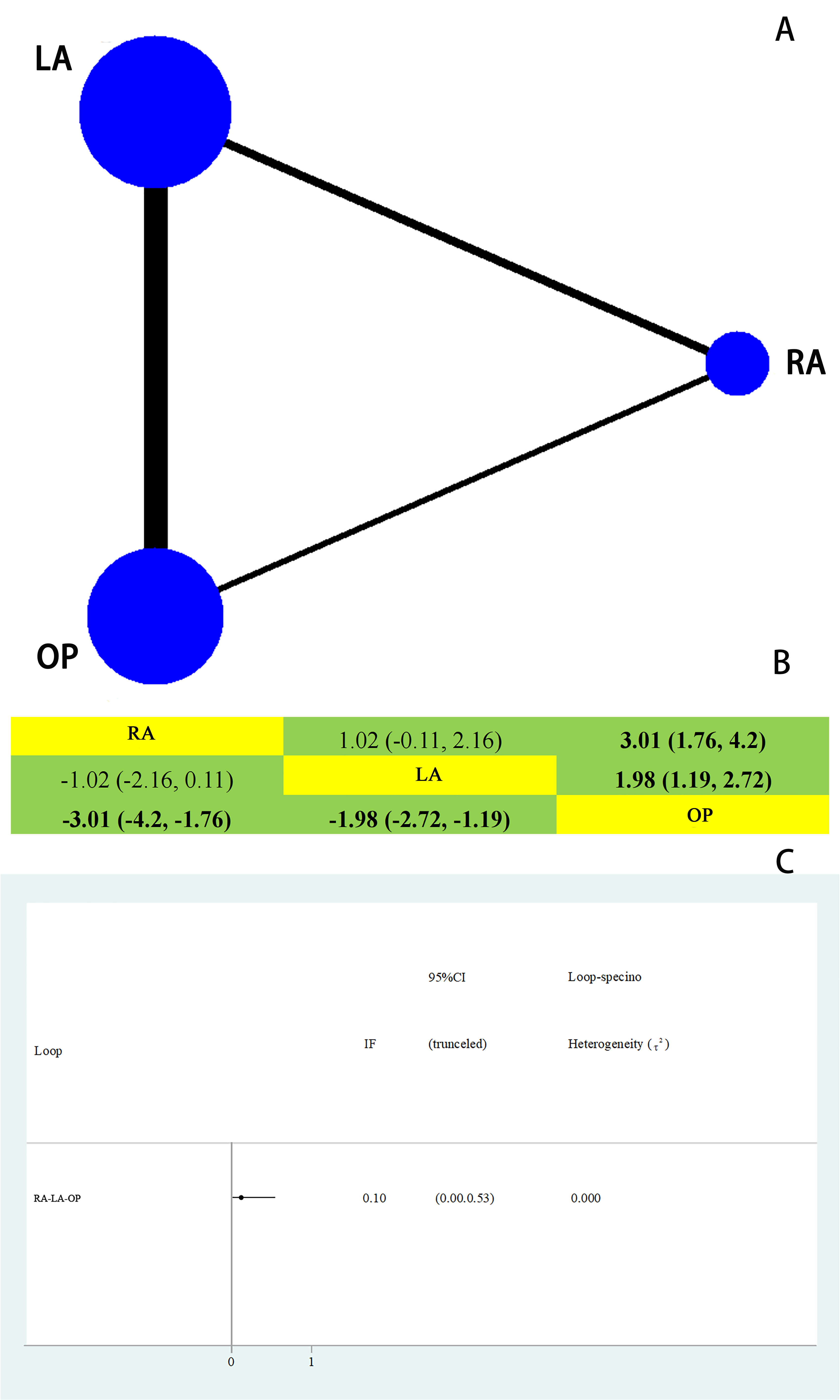

Supplement: Supplementary Figure S4 — Network Meta-analysis Results for Intraoperative Blood Loss Among the Three Surgical Methods. RA, robotic cyst excision and Roux-en-Y hepaticojejunostomy; LA, laparoscopic cyst excision and Roux-en-Y hepaticojejunostomy; OP, open cyst excision and Roux-en-Y hepaticojejunostomy. (A) Intraoperative blood loss mesh diagram; (B) Intraoperative blood loss mesh meta-analysis results; (C) Intraoperative blood loss consistency test chart. [file Image3.jpeg]

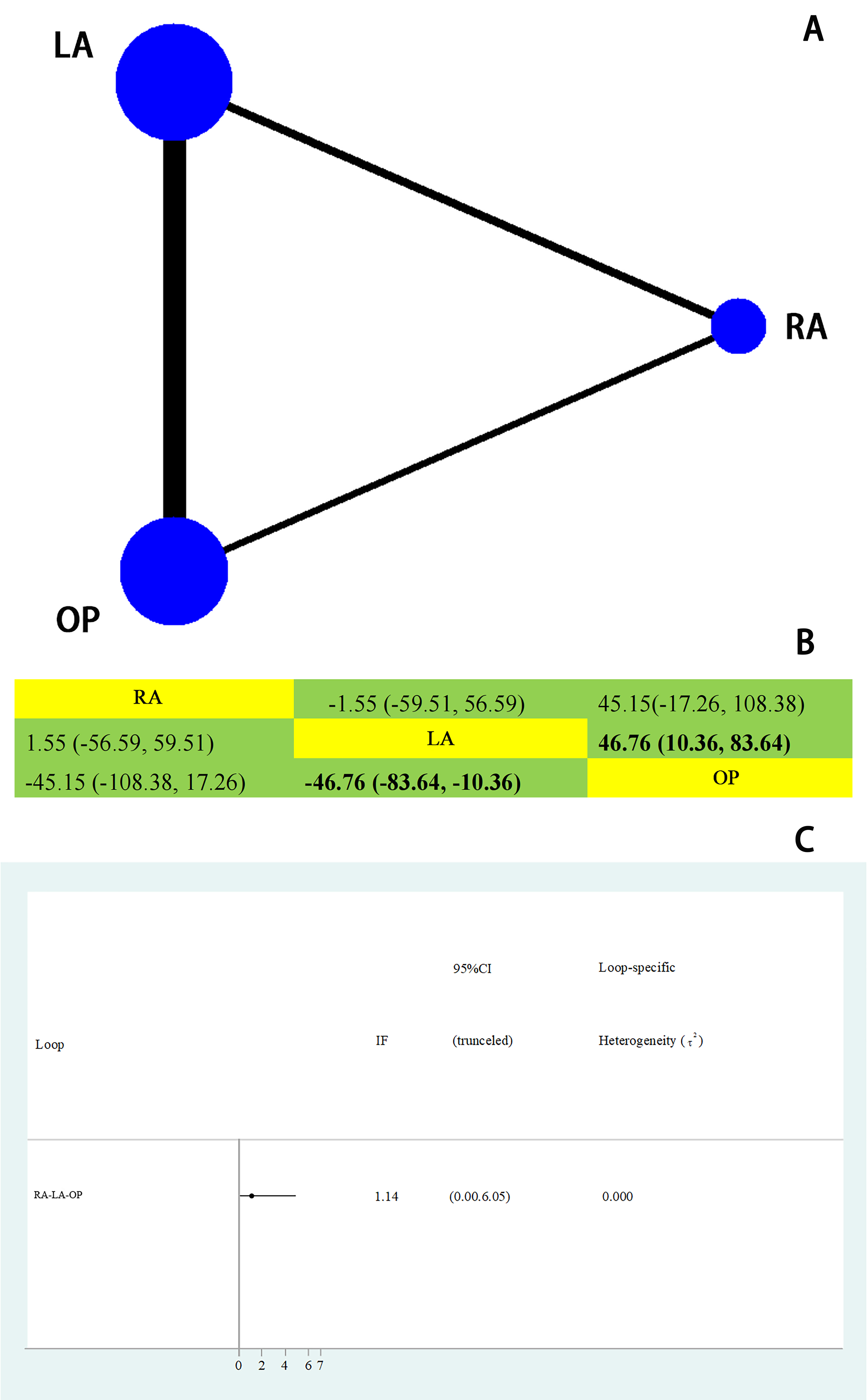

Supplement: Supplementary Figure S5 — Network Meta-analysis Results for Postoperative Bile Leakage Incidence Among the Three Surgical Methods. RA, robotic cyst excision and Roux-en-Y hepaticojejunostomy; LA, laparoscopic cyst excision and Roux-en-Y hepaticojejunostomy. OP, open cyst excision and Roux-en-Y hepaticojejunostomy. (A) Network diagram of postoperative biliary leakage incidence; (B) Results of mesh meta-analysis of postoperative biliary leakage; (C) Consistency test chart of postoperative biliary leakage. [file Image4.jpeg]

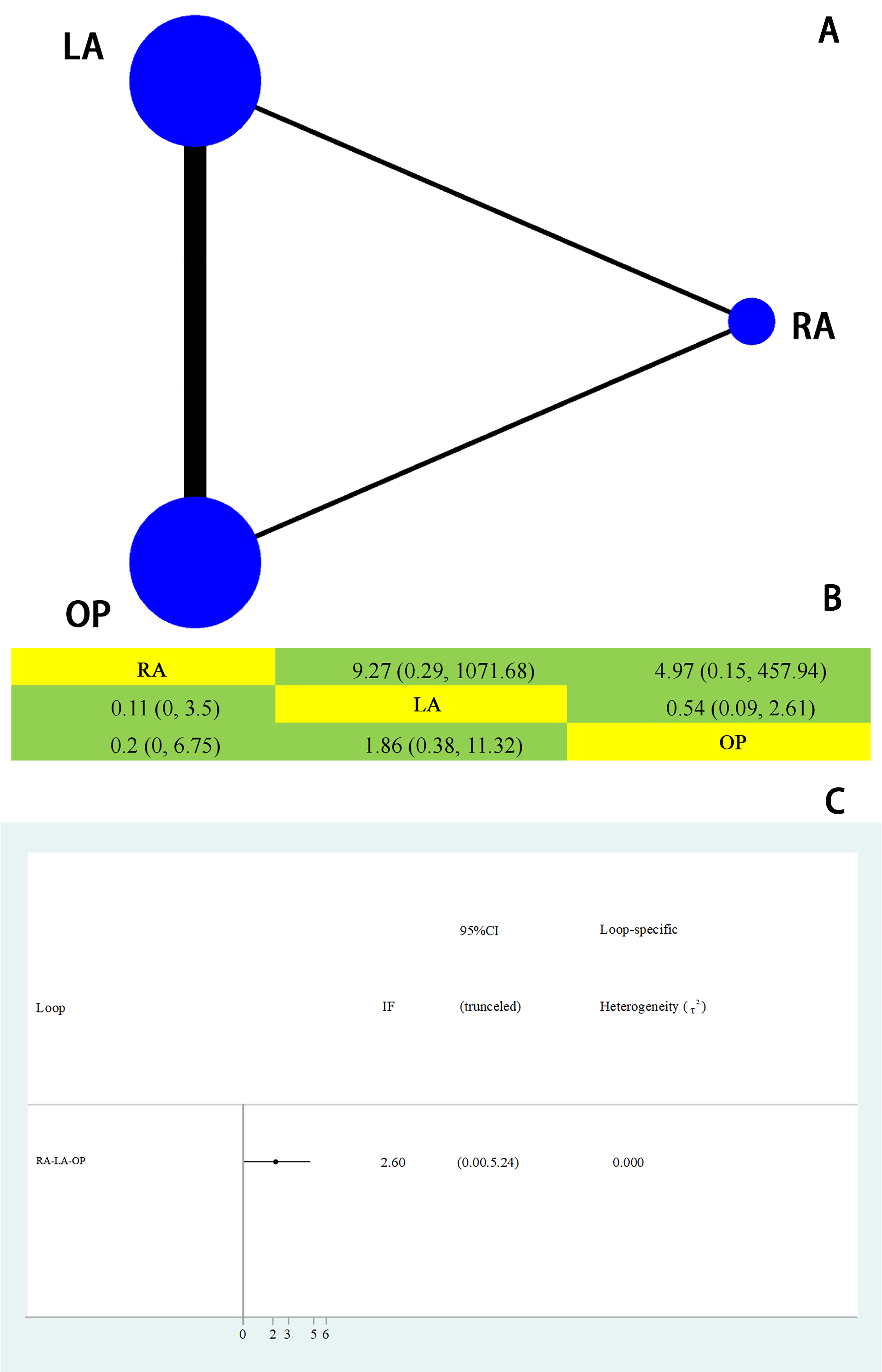

Supplement: Supplementary Figure S6 — Network Meta-analysis Results for Postoperative Intestinal Obstruction Incidence Among the Three Surgical Methods. RA, robotic cyst excision and Roux-en-Y hepaticojejunostomy; LA, laparoscopic cyst excision and Roux-en-Y hepaticojejunostomy; OP, open cyst excision and Roux-en-Y hepaticojejunostomy. (A) Reticular chart of postoperative ileus incidence; (B) Results of mesh meta-analysis of postoperative intestinal obstruction; (C) Consistency test chart of postoperative ileus incidence. [file Image5.jpeg]

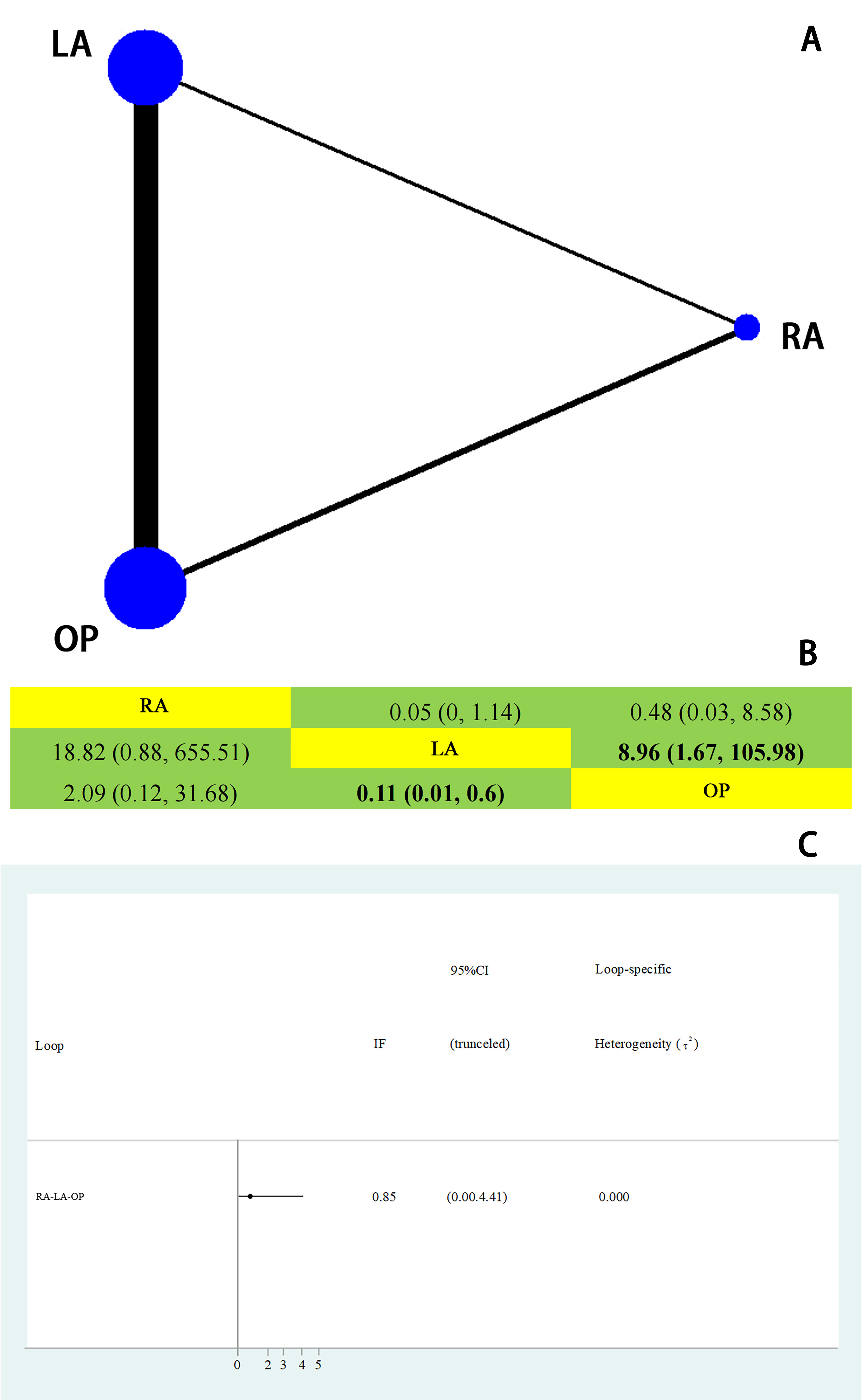

Supplement: Supplementary Figure S7 — Assessment of Publication Bias in Network Meta-analysis for Surgical Treatments of Congenital Choledochal Cyst. Note: RA, Robotic cyst excision and Roux-en-Y hepaticojejunostomy; LA, laparoscopic cyst excision and Roux-en-Y hepaticojejunostomy. OP, open cyst excision and Roux-en-Y hepaticojejunostomy. (A) Funnel plot assessing publication bias for surgical time comparisons among robotic-assisted, laparoscopic, and open surgeries. (B) Funnel plot evaluating publication bias for hospital stay comparisons across the three surgical methods. (C) Funnel plot analyzing publication bias for intraoperative blood loss comparisons among the surgical approaches. (D) Funnel plot depicting publication bias for postoperative bile leak incidence among the three surgical methods. (E) Funnel plot illustrating publication bias for postoperative bowel obstruction incidence among robotic-assisted, laparoscopic, and open surgeries. [file Image6.jpeg]

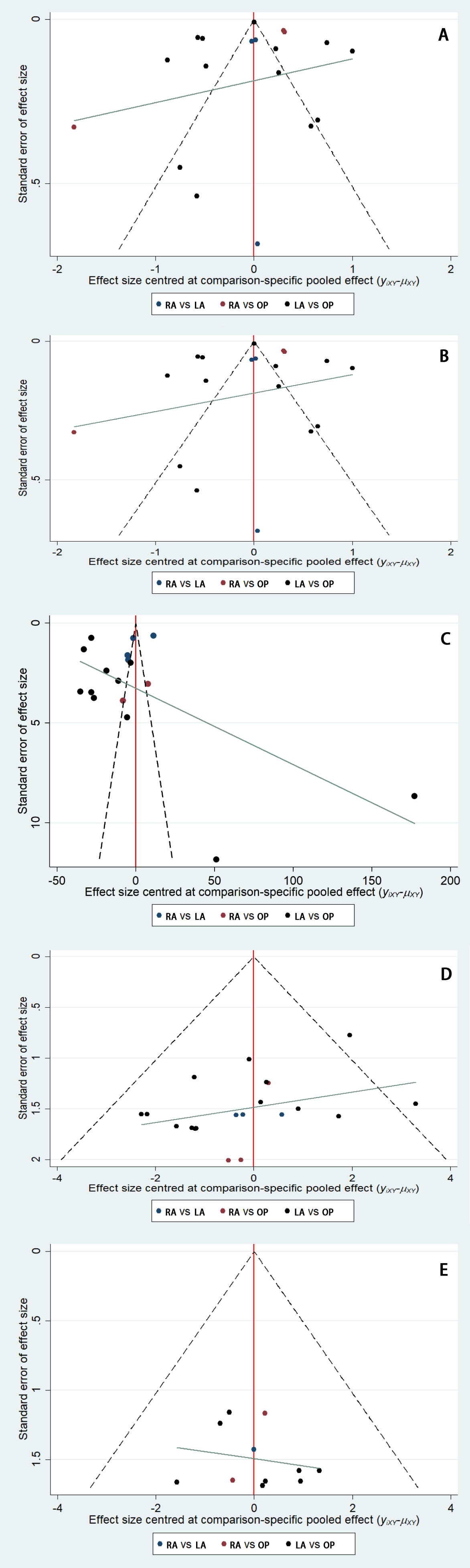

Supplement: Supplementary Figure S8 — Comparison of Network Meta-analysis Results for Surgery Duration Before and After Incorporating Retrospective Study Data. RA, robotic cyst excision and Roux-en-Y hepaticojejunostomy; LA, laparoscopic cyst excision and Roux-en-Y hepaticojejunostomy. OP, open cyst excision and Roux-en-Y hepaticojejunostomy. (A) Results of mesh meta-analysis of Surgery Duration; (B) surgical Duration consistency test chart. [file Image7.tif]

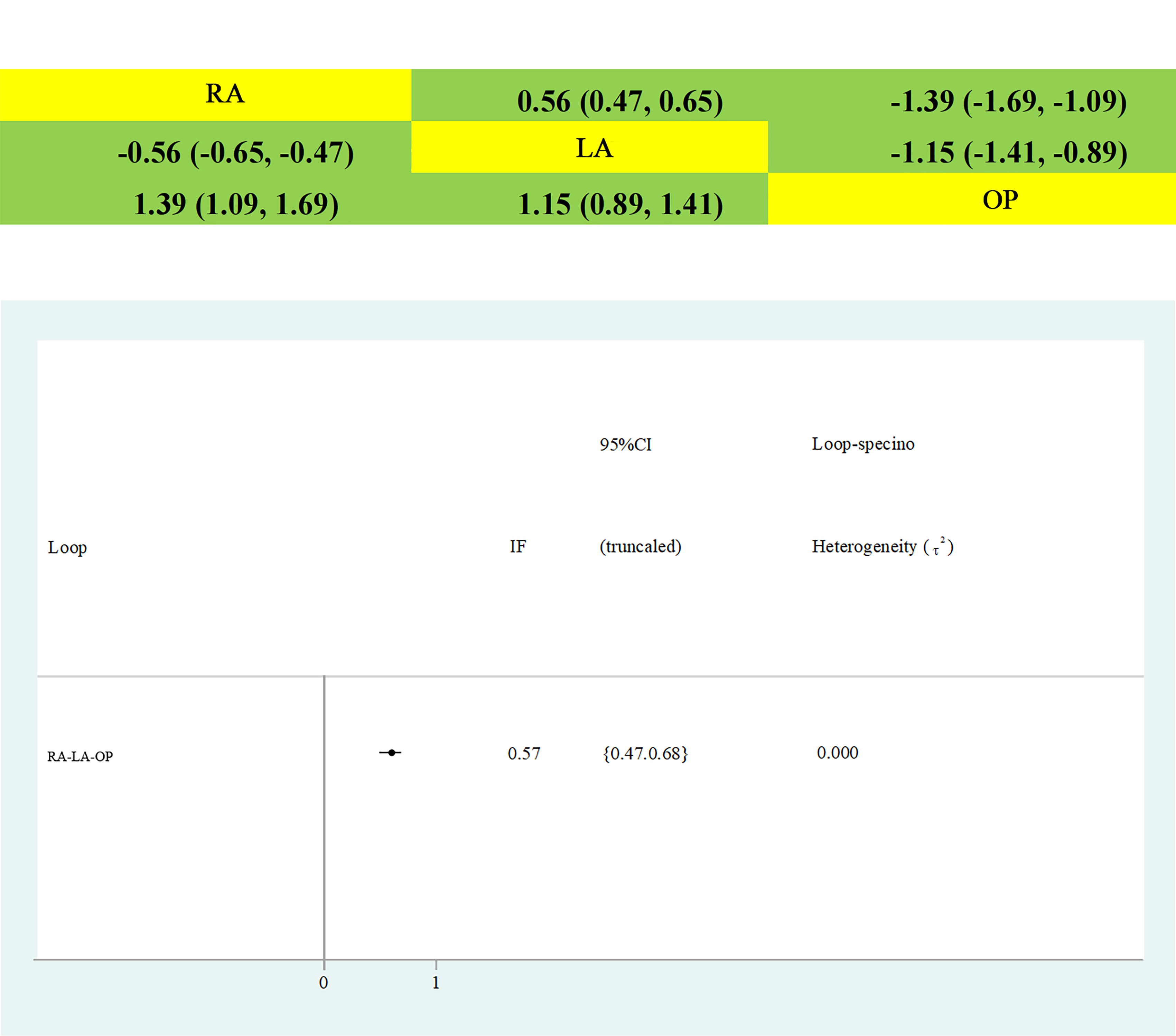

Supplement: Supplementary Figure S9 — Comparison of Network Meta-analysis Results for Hospitalization Duration Before and After Incorporating Retrospective Study Data. RA, robotic cyst excision and Roux-en-Y hepaticojejunostomy; LA, laparoscopic cyst excision and Roux-en-Y hepaticojejunostomy; OP, open cyst excision and Roux-en-Y hepaticojejunostomy. (A) Results of mesh meta-analysis of Hospitalization Duration; (B) Consistency test chart of Hospitalization Duration. [file Image8.jpeg]

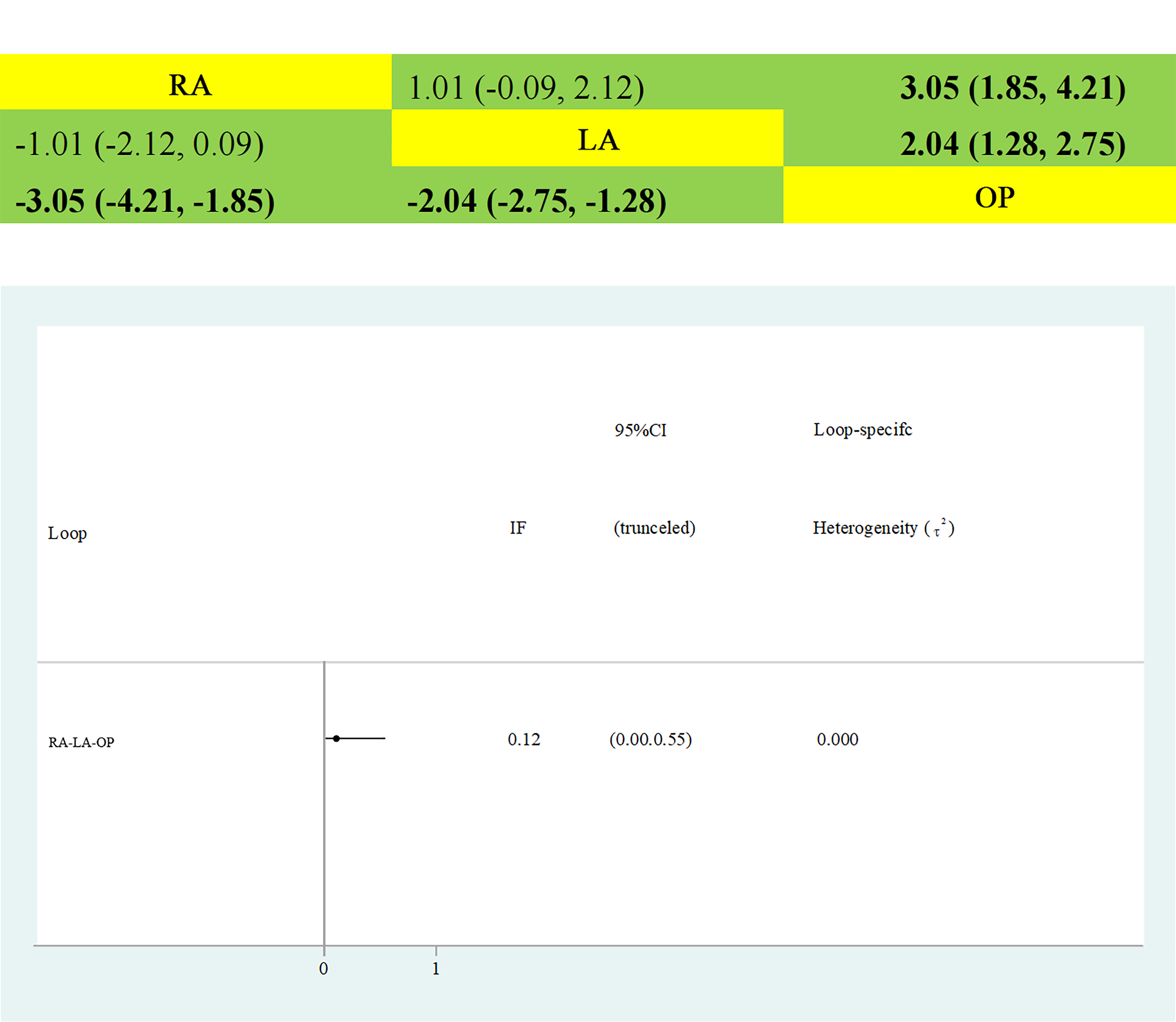

Supplement: Supplementary Figure S10 — Comparison of Network Meta-analysis Results for Intraoperative Blood Loss Before and After Incorporating Retrospective Study Data. RA, robotic cyst excision and Roux-en-Y hepaticojejunostomy; LA, laparoscopic cyst excision and Roux-en-Y hepaticojejunostomy. OP, open cyst excision and Roux-en-Y hepaticojejunostomy. (A) Intraoperative blood loss mesh meta-analysis results; (B) Intraoperative blood loss consistency test chart. [file Image9.tif]

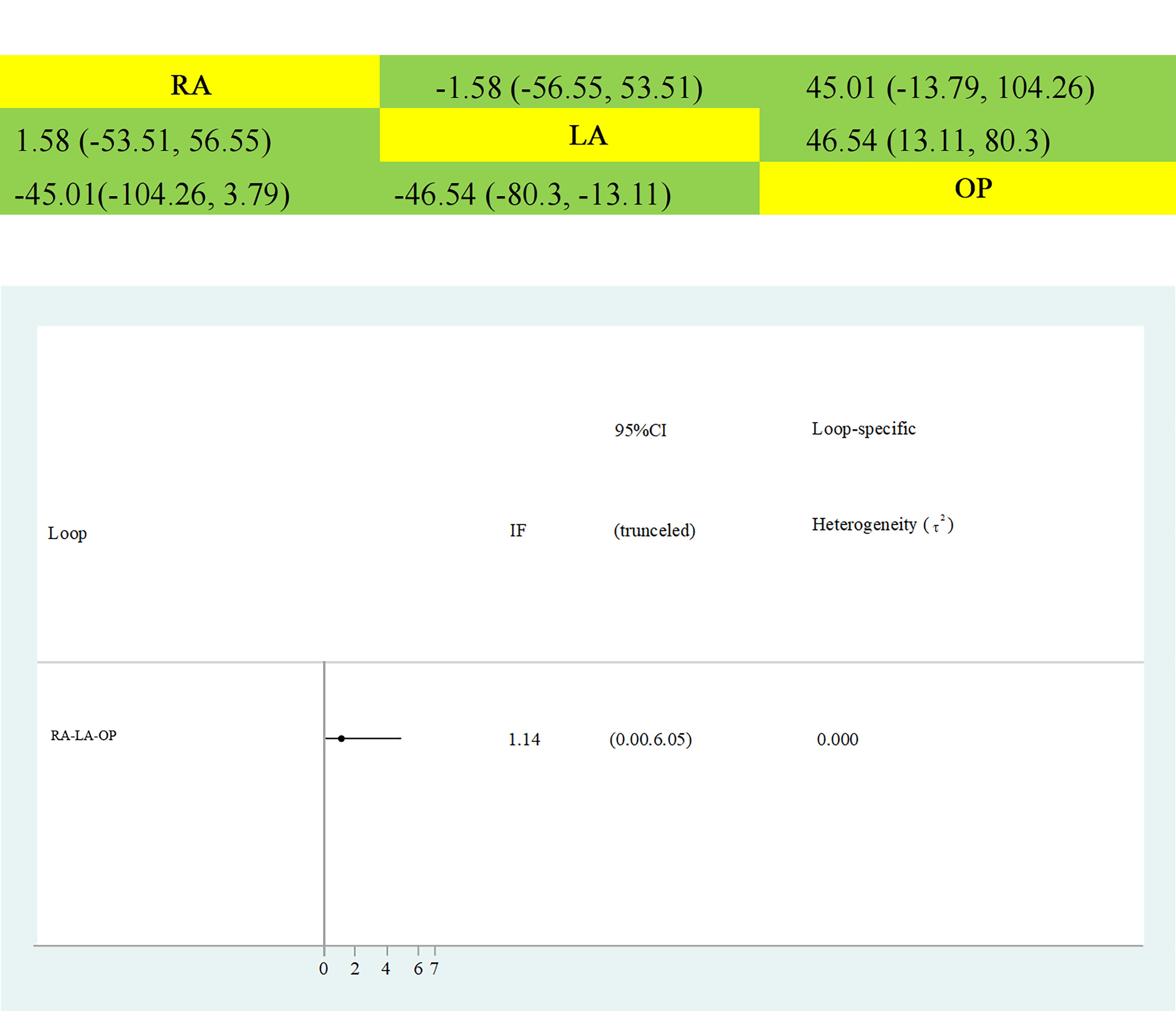

Supplement: Supplementary Figure S11 — Comparison of Network Meta-analysis Results for Postoperative Bile Leakage Incidence Before and After Incorporating Retrospective Study Data. RA, robotic cyst excision and Roux-en-Y hepaticojejunostomy; LA, laparoscopic cyst excision and Roux-en-Y hepaticojejunostomy; OP, open cyst excision and Roux-en-Y hepaticojejunostomy. (A) Results of mesh meta-analysis of postoperative biliary leakage; (B) Consistency test chart of postoperative biliary leakage. [file Image10.tif]

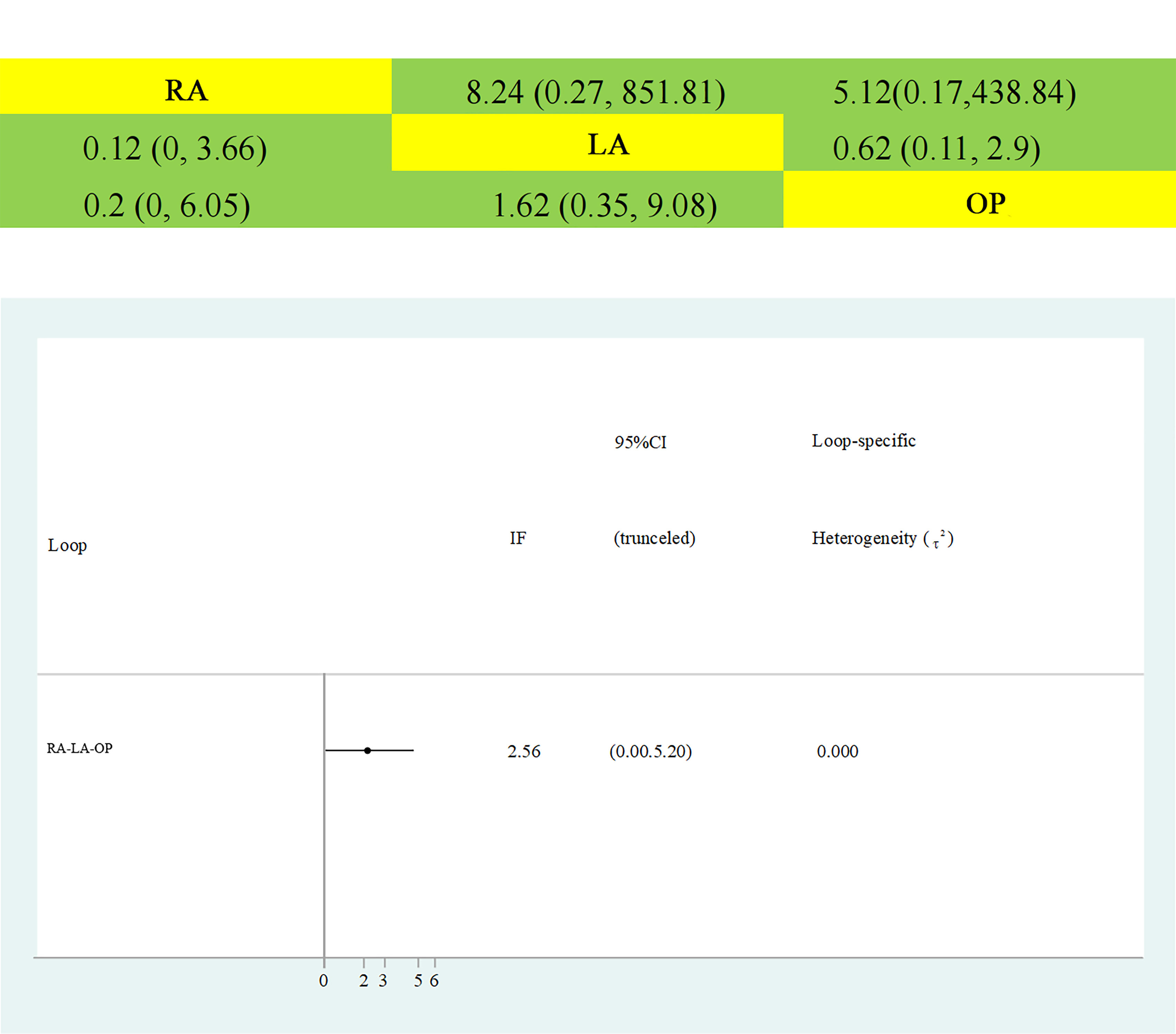

Supplement: Supplementary Figure S12 — Comparison of Network Meta-analysis Results for Postoperative Intestinal Obstruction Incidence Before and After Incorporating Retrospective Study Data. RA, robotic cyst excision and Roux-en-Y hepaticojejunostomy; LA, laparoscopic cyst excision and Roux-en-Y hepaticojejunostomy. OP, open cyst excision and Roux-en-Y hepaticojejunostomy. (A) Results of mesh meta-analysis of postoperative intestinal obstruction; (B) Consistency test chart of postoperative ileus incidence. [file Image11.jpeg]

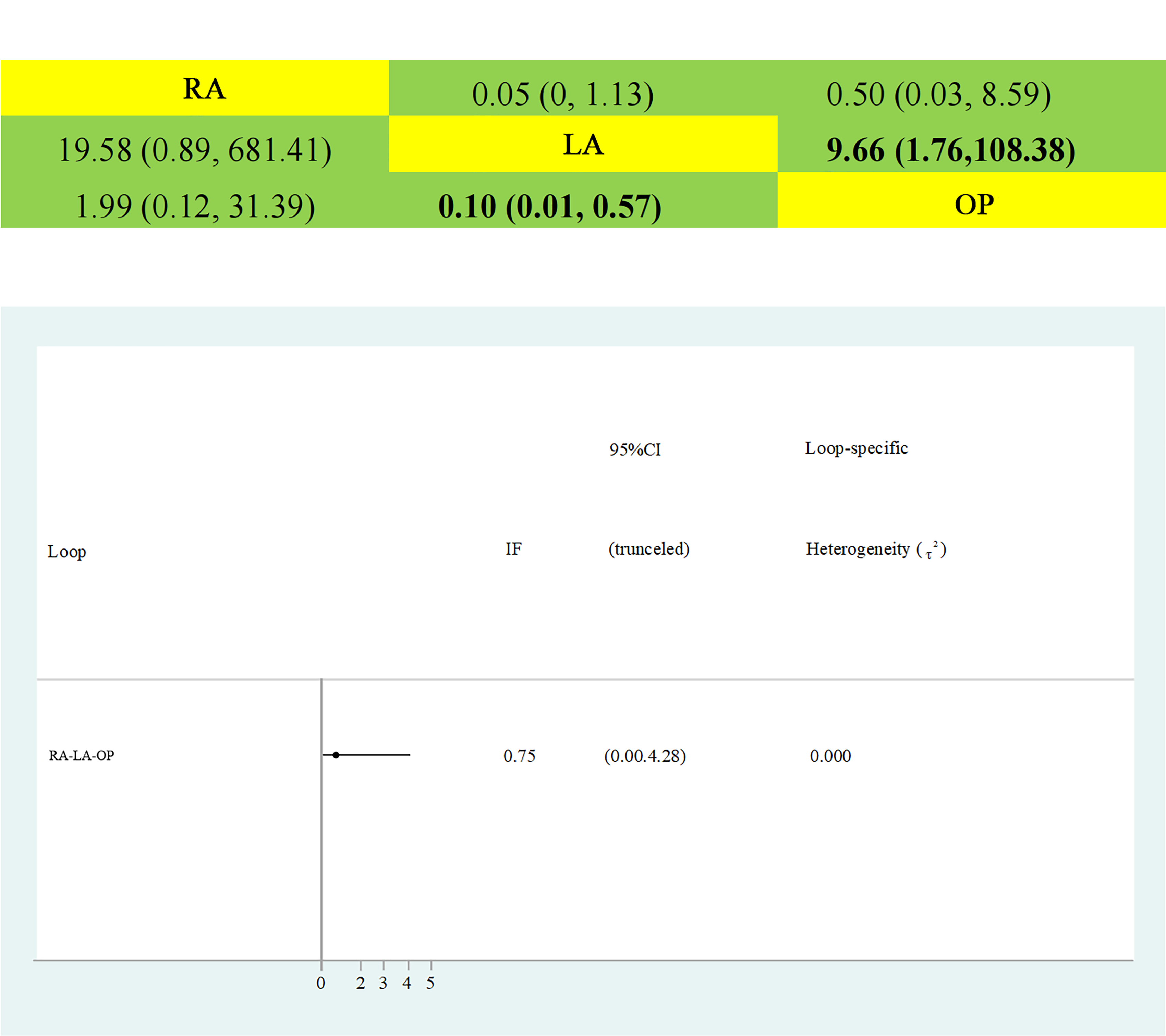

Supplement: Supplementary file 13 [file Image12.tif]
